# AMPK regulates homeostasis of invasion and viability in trophoblasts by redirecting glucose metabolism: Implications for pre‐eclampsia

**DOI:** 10.1111/cpr.13358

**Published:** 2022-12-08

**Authors:** Ping Xu, Yangxi Zheng, Jiujiang Liao, Mingyu Hu, Yike Yang, Baozhen Zhang, Mark D. Kilby, Huijia Fu, Yamin Liu, Fumei Zhang, Liling Xiong, Xiyao Liu, Huili Jin, Yue Wu, Jiayu Huang, Tingli Han, Li Wen, Rufei Gao, Yong Fu, Xiujun Fan, Hongbo Qi, Philip N. Baker, Chao Tong

**Affiliations:** ^1^ State Key Laboratory of Maternal and Fetal Medicine of Chongqing Municipality The First Affiliated Hospital of Chongqing Medical University Chongqing China; ^2^ Ministry of Education‐International Collaborative Laboratory of Reproduction and Development Chongqing Medical University Chongqing China; ^3^ Biochemistry and Molecular Biology University of Texas McGovern Medical School Houston Texas USA; ^4^ Department of Stem Cell Transplantation and Cell Therapy MD Anderson Cancer Center Houston Texas USA; ^5^ Department of Gynecology and Obstetrics Peking University Third Hospital Beijing China; ^6^ Shenzhen Institutes of Advanced Technology Chinese Academy of Sciences Shenzhen Guangdong China; ^7^ Institute of Metabolism and System Research University of Birmingham Edgbaston UK; ^8^ Department of Obstetrics Women and Children's Hospital of Chongqing Medical University Chongqing China; ^9^ Laboratory of Reproductive Biology, School of Public Health and Management Chongqing Medical University Chongqing China; ^10^ College of Life Sciences University of Leicester Leicester UK

## Abstract

Pre‐eclampsia (PE) is deemed an ischemia‐induced metabolic disorder of the placenta due to defective invasion of trophoblasts during placentation; thus, the driving role of metabolism in PE pathogenesis is largely ignored. Since trophoblasts undergo substantial glycolysis, this study aimed to investigate its function and regulatory mechanism by AMPK in PE development. Metabolomics analysis of PE placentas was performed by gas chromatography–mass spectrometry (GC–MS). Trophoblast‐specific *AMPKα1*‐deficient mouse placentas were generated to assess morphology. A mouse PE model was established by Reduced Uterine Perfusion Pressure, and placental AMPK was modulated by nanoparticle‐delivered A769662. Trophoblast glucose uptake was measured by 2‐NBDG and 2‐deoxy‐d‐[^3^H] glucose uptake assays. Cellular metabolism was investigated by the Seahorse assay and GC–MS.PE complicated trophoblasts are associated with AMPK hyperactivation due not to energy deficiency. Thereafter, AMPK activation during placentation exacerbated PE manifestations but alleviated cell death in the placenta. AMPK activation in trophoblasts contributed to GLUT3 translocation and subsequent glucose metabolism, which were redirected into gluconeogenesis, resulting in deposition of glycogen and accumulation of phosphoenolpyruvate; the latter enhanced viability but compromised trophoblast invasion. However, ablation of AMPK in the mouse placenta resulted in decreased glycogen deposition and structural malformation. These data reveal a novel homeostasis between invasiveness and viability in trophoblasts, which is mechanistically relevant for switching between the ‘go’ and ‘grow’ cellular programs.

## Graphical abstract

Pre‐eclampsia (PE) is associated with trophoblast AMPK hyperactivation, presumably due to LKB1 phosphorylation, and glucose uptake is consequently increased via trafficking of GLUT3 from the cytosol to the plasma membrane. Such translocation enhances glycolytic flux and redirects glucose metabolic intermediates into gluconeogenesis, resulting in PEP accumulation, which not only benefits cell survival but also suppresses invasion by repressing MMPs, and thus in turn modulates switching between the ‘go’ and ‘grow’ cellular programs.

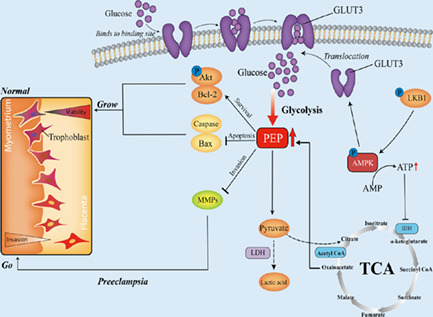

## 1 BACKGROUND

Preeclampsia (PE) is a pregnancy complication of hypertension that causes morbidity and mortality in both the mother and fetus^1^ and is characterized by increased levels of certain molecules in the maternal circulation that lead to maternal multiorgan dysfunction. Numerous studies have suggested that insufficient invasion capability of trophoblasts during the first trimester may play an important role in inadequate vascular remodelling of the placenta, which ultimately leads to a hypoxic intrauterine environment.^2^ Thus, compromised trophoblast invasiveness has been assumed to be one of the direct pathogenic drivers of PE.

AMP‐activated protein kinase (AMPK), the master switch of energy metabolism,^3^, ^4^ has profound influences in various disease conditions,^5^ especially ischemia.^6^ AMPK knockdown (KD) in both placental labyrinthine progenitor cells and differentiated placental trophoblasts not only impacts cellular metabolism^7^ but also alters the cell growth rate and nutrient transport.^8^ However, the involvement of AMPK in PE pathogenesis remains largely unknown.

The glucose transporter (GLUT) protein family is encoded by *SLC2* genes and transports monosaccharides, polyols, and other small carbon compounds across the lipid bilayer membranes of eukaryotic cells when expressed ectopically.^9^ Regulation of different GLUTs by AMPK has been reported in various tissues and cells.^10^ To date, 14 GLUTs with temporal and spatial expression patterns have been identified in humans.^11^ Among them, GLUT1, GLUT3, GLUT4 and recently, GLUT9 and GLUT12 have been found in the placenta or trophoblasts.^12^, ^13^, ^14^ Interestingly, upregulation of GLUT expression appears to be associated with an aggressive cancer phenotype,^15^, ^16^ probably due to enhanced glucose uptake and metabolism.^17^ Previous findings have also suggested that astrocytes increase GLUT3 expression to enhance glucose uptake in response to hypoxia, even though GLUT1 is the predominant GLUT^18^; this finding indicates that coexpressed GLUT isoforms may facilitate glucose uptake cooperatively or individually under different conditions. In this study, we investigated the role of AMPK in the regulation of trophoblast invasion and determined the involvement of GLUT3 in AMPK‐regulated glucose metabolism in trophoblasts. These findings may expand our understanding of PE pathogenesis.

## 2 METHODS

### 2.1 Subject recruitment and placenta sampling

Fifteen PE patients and 15 patients with normal pregnancies admitted to the Department of Obstetrics at The First Affiliated Hospital of Chongqing Medical University for caesarean deliveries from October 2016 to October 2017 were randomly recruited for this study. The exclusion criteria included gestational diabetes mellitus, cardiovascular diseases, collagen disorder, chronic renal disease, chronic hypertension, intrahepatic cholestasis of pregnancy, anaemia, infectious disease, drug use or other obstetric complications. The controls were patients with normotensive pregnancies who underwent caesarean section (C‐section) due to breech presentation or patient request. PE was diagnosed in individuals with a systolic blood pressure of 140 mmHg or greater, a diastolic blood pressure of 90 mmHg or greater, or both, according to the guidelines of the American College of Obstetrics and Gynaecology (ACOG). The clinical characteristics of the study populations are summarized in Table S1. Five to ten 5‐mm^3^ pieces of placental tissue were collected from the side of each patient near the insertion point of the umbilical cord immediately after the C‐section, rinsed in ice‐cold 0.9% sterile normal saline (NS) three times to remove blood clots, blotted dry and frozen in liquid nitrogen until further use.

### 2.2 Isolation of primary human trophoblasts

Healthy and PE‐complicated term placentas obtained soon after C‐section were used for the isolation of primary human trophoblasts (PHTs) as previously described.^19^ Briefly, 50 g of placental tissue was cut into small pieces and washed with PBS at room temperature. The tissue was enzymatically digested with sequential trypsin‐DNase digestions at 0.25%. Then, a discontinuous gradient of Percoll (5%–60%, in four steps; Sigma–Aldrich, Germany) was applied to remove red blood cells and cell debris. The middle‐layer cells were collected and washed with DMEM/F12 (Gibco). Then, the cells were resuspended in DMEM/F12 supplemented with 5 μg/ml insulin (Sigma–Aldrich, Germany), 10 μg/ml transferrin (Sigma–Aldrich, Germany), 20 nM sodium selenite (Sigma–Aldrich, Germany), 5 ng/ml EGF (Sigma–Aldrich, Germany), 400 U/L hCG (Abcam, UK), 10% FBS (Gibco), 1% penicillin/streptomycin (Gibco), 50 μg/ml gentamycin (Gibco) and 0.25 μg/ml amphotericin (Gibco). The PHTs were further verified by flow cytometry with anti‐CD31 (Thermo Fisher) and anti‐CK7 (Thermo Fisher) antibodies (Figure S1A).

### 2.3 Villous explant outgrowth assay

First‐trimester placental villus samples were obtained from healthy women undergoing elective surgical termination of their pregnancies between 6 and 9 weeks of gestation. These samples were kept in cold sterile saline and dissected into explants 1–2 mm in diameter. Before implantation of the explants, growth factor‐reduced Matrigel (Corning) mixed 1:9 with serum‐free DMEM/F12 medium (Life Technologies) was added to 48‐well culture plates (BIOFIL, China) and incubated at 37°C and 5% CO_2_ for 8 h to gel. Then, 600 μl of DMEM/F12 medium with 10% FBS, 100 U/ml penicillin, 100 μg/ml streptomycin and 0.1% ethidium homodimer‐2 (Thermo Fisher) was added to the 48‐well culture plates. The explants were cultured at 37°C and 5% CO_2_ for 15 h and then observed and recorded by an EVOS FL Auto microscope (Life Technologies). Only explants with successful initiation of trophoblast outgrowth were selected. DMSO (0.3%), 200 μM AICAR, lentiviruses carrying sh*SLC2A3* with or without AICAR and universal control shRNA were added. The explants were incubated for 48 h and recorded. The transfection efficiency was monitored based on the expression of green fluorescent protein (GFP). Explants from the same placental villi were paired for the control and experimental groups. For each explant, at least four distances from the cell column base to the tip of the outgrowth were measured using ImageJ Software (National Institutes of Health). All experiments were repeated three times.

### 2.4 Measurement of ATP levels in placental tissue

ATP levels were measured using a commercial ATP assay kit (Abcam, UK). In brief, placental tissue was homogenized with a Dounce homogenizer and then centrifuged for 5 min at 4°C at 13,000*g*. The supernatant was transferred to a new tube, and interfering enzymes were removed using a Deproteinizing Sample Preparation Kit (Abcam, UK). The supernatants were then collected for ATP measurement using an ATP assay kit according to the manufacturer's instructions. A spectrophotometer (Thermo Fisher, Finland) was used to read the fluorescence at an excitation wavelength of 535 nm and an emission wavelength of 587 nm.

### 2.5 Measurement of AMP levels in the placenta

AMP levels were measured using a commercial AMP assay kit (Bio Vision). In brief, placental tissue was rapidly homogenized in old AMP assay buffer and then centrifuged for 10 min at 4°C at 10,000*g*. Supernatants were then collected for BCA and AMP measurement according to the manufacturer's instructions. The absorbance at 570 nm is proportional to the amount of AMP present in the samples.

### 2.6 Glucose level measurement

One hundred milligrams of placental tissue was homogenized and then incubated at 95°C for 10 min. Once cooled, the tissue was centrifuged for 10 min at 25°C at 8000*g*. The supernatant was collected for glucose level measurement using a glucose assay kit (Solarbio, China) according to the manufacturer's instructions; the absorbance was read at 505 nm using a microplate reader (Thermo Fisher).

### 2.7 Establishment of an in vivo PE mouse model by reduced uterine perfusion pressure

A reduced uterine perfusion pressure (RUPP) mouse model was established by a skilled researcher as previously reported with modifications.^20^, ^21^, ^22^, ^23^ Briefly, 8‐ to 10‐week‐old C57/BL6J mice were obtained from the Experimental Animal Center of Chongqing Medical University. The animals were maintained under a 12‐h light/12‐h dark cycle in a controlled environment with access to water ad libitum. Observation of a vaginal sperm plug in the morning after overnight mating was designated gestational day (GD) 0.5. On GD 13.5, the pregnant mice were anaesthetised with isoflurane with an animal anaesthesia apparatus (Surjivet) coupled with a rodent ventilator (Harvard Apparatus). A 2‐cm incision on the abdomen that included skin and the peritoneum was made as close as possible to the linea alba. Next, four silver clips (80 μm) were placed around the arterial and venous branches of the vascular arcade of both the ovarian and uterine vessels. No clips were used in the sham group. The peritoneum and skin were sutured with 7‐0 and 5‐0 silk (Lingqiao, China), respectively. Blood pressure was measured by tail‐cuff plethysmography (Visitech System) in conscious mice, and hemodynamics were assessed with a Vivid E9 ultrasound system (GE Healthcare) and an i13L ultrasonic probe (GE Healthcare) under inhaled anaesthesia. All phenotypic measures were conducted in a blinded group allocation.

### 2.8 Trophoblast‐specific AMPK activation in mice

The tissue specificity of drug delivery was examined by administration of placental chondroitin sulfate A‐binding peptide (plCSA)‐conjugated NPs (plCSA‐NPs) loaded with indocyanine green (Sigma–Aldrich, Germany) in pregnant C57/BL6J mice randomly at GD 14.5 through tail vein injection. Mice were then scanned using an IVIS Spectrum instrument (PerkinElmer) 30 min after injection. plCSA‐NPs loaded with A769662 (plCSA‐A769662) (5 μM) or plCSA‐NPs loaded with vehicle (DMSO) were administered to C57/BL6J mice three times either at GD 8.5, GD 10.5 and GD 12.5 or in late gestation immediately after sham or RUPP operation, namely, at GD 13.5, GD 15.5 and GD 17.5. Urine and blood were collected at GD 18.5.

### 2.9 Haematoxylin and eosin staining and periodic acid–Schiff staining

For haematoxylin and eosin (H&E) staining, sections were deparaffinized, rehydrated, incubated with haematoxylin for 1 min, washed by floating in tap water for 5 min and stained with eosin (Sigma–Aldrich) for 1 min. Ten images of each section were captured with an EVOS microscope (Life Technologies). Periodic acid–Schiff (PAS) staining was carried out with a commercial staining kit (Bioservice, China). In brief, after deparaffinization and rehydration, sections were incubated with 0.5% periodic acid solution for 5 min, stained with Schiff's reagent for 15 min, and counterstained with haematoxylin solution for 2 min. The sections were rinsed three times with tap water after each step. Ten images of each section were captured with an EVOS microscope (Life Technologies).

### 2.10 Transgenic mice

*AMPKα1* ^*f/f*^ mice possessing *loxP* sites flanking exon 3 of the protein kinase AMP‐activated alpha 1 catalytic subunit (*Prkaa1*) gene were generated by Beijing Biocytogen Co., Ltd. *Tpbpar/Adaf‐AdaP‐Cre* ^+^ mice (*Ada‐Cre* ^+^) were obtained as a gift from Prof. Xin Ni (Xiangya Hospital, Central South University). *This Cre* transgene expression occurred throughout the placenta but not in maternal organs or in the fetus.^24^ The placenta‐specific deletion of *AMPKα1* was generated by crossing mice homozygous for the floxed *AMPKα1* allele with mice expressing *Ada‐Cre*. Then, 10‐ to 12‐week‐old *AMPKα1* ^*f/f*^ *Ada*‐Cre^+^ males were mated with age‐matched *AMPKα1* ^*f/f*^ females. The primers used for genotyping are listed in Table S4.

### 2.11 Trophoblast‐targeted nanoparticles

Trophoblast‐targeted nanoparticles (NPs) were synthesized as previously reported^25^, ^26^ and loaded with the AMPK‐specific agonist A769662 (Selleckchem). To determine the encapsulation efficiency (EE) and drug loading efficiency (LE) of A769662 in the NPs, newly synthesized NPs were isolated from the aqueous suspensions before ultrafiltration using a Beckman Optima™ MAX‐XP ultracentrifuge (34,000*g*, 30 min) (Beckman). The nonentrapped A769662 in the supernatant was quantified using fluorescence spectroscopy (Edinburgh Instruments Ltd., UK) with emission at 545 nm and excitation at 300 nm. The EE and LE were calculated as follows: EE = (weight of loaded A769662)/(weight of initially added A769662) × 100% and LE = (weight of loaded A769662)/(total weight of NPs) × 100%.

### 2.12 Serum sFlt‐1 measurement

Blood was drawn by cardiac puncture when sacrificing the mice and then centrifuged for 20 min at 4°C at 1000*g*. The serum was transferred to a new tube and then diluted 1:1 with sample dilution buffer. One hundred microliters of each diluted sample was used to detect the serum sFlt‐1 concentration with a Mouse VEGFR1/FLT1 ELISA Kit (Elabscience, China) following the manufacturer's instructions. The absorbance was read at 450 nm by using a microplate reader (Thermo Fisher).

### 2.13 Mouse urinary albumin detection

Urine samples were centrifuged for 5 min at 4°C at 10,000 rpm to remove insoluble materials. The samples were then diluted 1:1000 with dilution buffer. Fifty microliters of each diluted sample was used to detect the mouse urinary albumin concentration with a Mouse Urinary Albumin Detection Kit (Chondrex) following the manufacturer's instructions. The absorbance was read at 490 nm using a microplate reader (Thermo Fisher).

### 2.14 TUNEL staining

Frozen slides were created for mouse uteroplacental units collected at GD 8.5 and GD 11.5 and incubated with the reagents in an In Situ Cell Death Detection Kit‐TUNEL, TMR Red (Roche, Schweiz), at 37°C for 1 h following the manufacturer's instructions. The stained sections were mounted using mounting medium containing DAPI (Vector Laboratories) and examined by confocal microscopy (Zeiss, Germany). HTR8/SVneo cells were plated in 48‐well plates, cultured until reaching 60%–70% confluence, and then treated with 0.4 mM phosphoenolpyruvate (PEP; Sigma–Aldrich) for 24 h. Then, a TUNEL kit (Beyotime, China) was used to measure the apoptosis rate according to the manufacturer's instructions. DNase I (Sigma–Aldrich) pretreatment was performed for the positive control samples. Images were captured by an EVOS microscope (Life Technologies).

### 2.15 Cell line

The human HTR8/SVneo trophoblast line was purchased from the American Type Culture Collection (ATCC). The cells were cultured in RPMI 1640 medium (Gibco) supplemented with 10% fetal bovine serum (FBS, Gibco) and 1% penicillin/streptomycin (Beyotime, China) at 37°C in 5% CO_2_/20% O_2_.

### 2.16 shRNA transfection

Three shRNAs targeting GLUT3 (*SLC2A3* gene) and a scrambled negative control (NC) shRNA (shNC) were designed according to the GLUT3 mRNA sequence (GenBank ID: 6515) (Table S2). BLAST analyses did not show nonspecific interactions of either sh*SLC2A3* or shNC with other mRNA transcripts. Lentiviral particles were generated in 293FT cells via cotransfection with the packaging vector LV3‐GFP‐Puro and Lipofectamine 2000 (Invitrogen). The shRNAs were transfected into HTR8/SVneo trophoblasts with 5 μg/ml polybrene (Sigma–Aldrich) following the manufacturer's protocol. Transfected cells were then screened with 1 μg/ml puromycin (Gibco).

### 2.17 2‐NBDG uptake assay

HTR8/SVneo cells that grew to 80%–90% confluence in petri dishes were washed three times with prewarmed PBS and incubated for 30 min at 37°C with glucose‐free 1640 medium (Gibco) containing 10% FBS and 100 μm 2‐NBDG (Invitrogen). Then, the cells were washed in prewarmed PBS three times. Images were captured by an EVOS fluorescence microscope (Life Technologies). Then, the cells were harvested by digestion with 0.25% trypsin–EDTA solution (Gibco) for 1 min. After centrifugation at 1000 rpm for 5 min, the cells were resuspended in 500 μl of PBS. Fluorescence intensity was measured by a flow cytometer (2‐NBDG λ Ex/Em = 485/535 nm).

### 2.18 2‐Deoxy‐D‐[ ^3^H] glucose uptake assay

The 2‐Deoxy‐d‐[3H] glucose uptake by HTR8/SVneo cells was determined as previously described.^27^ WT, shNC and GLUT3‐KD cells were seeded in 12‐well plates at a density of 8 × 10^4^ cells/well and cultured until they reached 70% confluence. Prior to experimentation, cells were serum deprived for 2 h in glucose‐free medium and preincubated for 20 min at 37°C in 1 ml of warm Krebs–Ringer bicarbonate (KRH) buffer (25 mM HEPES‐NaOH [pH 7.4], 120 mM NaCl, 5 mM KCl, 1.2 mM MgSO_4_, 1.3 mM CaCl_2_, 1.3 mM KH_2_PO_4_) to acclimatize to conditions and deplete cellular glucose. Glucose uptake was initiated by the addition of 0.5 ml of KRH buffer containing 0.25 μCi/ml 2‐deoxy‐d‐[^3^H] glucose (Perkin Elmer) and 50 μM 2‐deoxy‐d‐glucose followed by 1 h of incubation at 37°C. Glucose uptake was stopped by removing the buffer and washing the cells three times with ice‐cold KRH buffer. The cells were lysed (0.5 M NaOH, 0.1% SDS), and the amount of labelled glucose taken up was determined with a Multi‐Purpose Scintillation Counter (Beckman Coulter). The total protein concentration was determined by the Bradford method using BSA as the standard. Radioactive glucose uptake is expressed as Bq/mg protein.

### 2.19 5‐Ethynyl‐20‐deoxyuridine DNA synthesis assay

The HTR8/SVneo cells were plated in 96‐well plates and cultured until reaching 70% confluence. An 5‐ethynyl‐20‐deoxyuridine (EdU) detection kit (RiboBio, China) was used to measure cell proliferation activity according to the manufacturer's instructions. Cells were first treated with 50 μmol/L EdU at 37°C for 2 h, fixed in 4% paraformaldehyde for 30 min, incubated with 2 mg/ml glycine for 5 min and 0.5% Triton X‐100 in PBS for another 10 min, and then stained with 1× Apollo reaction cocktail for 30 min at room temperature. Finally, cell nuclei were counterstained with Hoechst 33342. Images were captured by an EVOS fluorescence microscope (Life Technologies). The proliferation rate was calculated by the ratio of EdU‐positive cells to DAPI‐positive cells.

### 2.20 Gas chromatography–mass spectrometry‐based metabolomics analysis

Five‐millimetre pieces of placental tissue were rinsed in ice‐cold 0.9% sterile normal saline (NS) three times to remove blood clots, blotted dry and frozen in liquid nitrogen until further use. HTR8/SVneo cells were washed three times with ice‐cold PBS. PBS was aspirated from the cell surface, and liquid nitrogen was then added to quench cellular metabolism. Afterward, metabolites were extracted with methanol/toluene premixed with internal standards, nonadecanoic acid (50 μg/ml) and tridecanoic acid (50 μg/ml) (Nu‐Chek Prep Inc.). The metabolite extracts were collected in a glass tube, and acetyl chloride solution was added for fatty acid derivatization. After the tubes were sealed completely, the samples were heated and stirred at 100°C for 1 h. During this incubation time, the nonesterified fatty acids (NEFAs) were derivatized to their fatty acid methyl esters (FAMEs). After aqueous potassium carbonate solution was added to each sample for neutralization, the organic layer was collected and analysed by GC–MS. A GC system (Agilent Technology) coupled to a mass selective detector (Agilent Technology) was used to analyse fatty acid derivatives. A RESTEK Rtx®‐2330 column (90% biscyano‐propyl/10% phenylcyanopropyl polysiloxane) was installed to separate fatty acid isomers. The GC oven was set to 45°C, and the temperature was held for 2 min. The temperature was then increased to 215°C at a rate of 10°C/min and held for 35 min. The temperature was further elevated to 250°C at a rate of 40°C/min and held for 10 min. The quadrupole mass spectrometer was equipped with an electron ionization (EI) source operating at 70 eV. The instrument was set at a scan rate of 3.8 scans/sector with a mass range between 41 u and 420 u. The interface temperature was set to 230°C, and the quadrupole temperature was 150°C. A 14.5‐min solvent delay was applied to remove solvent interference.

After acquiring the GC–MS raw data, the GC chromatogram was deconvoluted. An Automated Mass Spectral Deconvolution and Identification System (AMDIS, version 2.72) was used for metabolite identification. The abundance of the highest ion for each identified metabolite was further extracted by our in‐house R software package MassOmics. The internal standards and total useful ion count were used to normalize the abundance of identified metabolites to correct for instrumental and human variabilities. Pairwise comparisons for the assessment of significant differences between experimental groups were performed via Tukey's HSD test in R. Heatmaps and receiver operating characteristic (ROC) curves were generated by the ggplot2 and pROC R packages, respectively.

### 2.21 Seahorse metabolic assay

To test the effects of different drugs on cellular bioenergetics in HTR8/SVneo cells, an XFe96 Extracellular Flux Analyser (Agilent Technologies) was utilized. The assay medium was prepared immediately before the experiment. The medium consisted of Seahorse XF Base Medium without phenol red with 2 mM glutamine (Gibco), 10 mM glucose (Gibco), 1 mM pyruvate (Sigma–Aldrich), and 5.0 mM HEPES (Agilent Technologies). The pH was adjusted to 7.4 at 37°C, and a 0.2‐μm filter was used for sterilization. WT HTR8/SVneo cells and GLUT3‐KD HTR8/SVneo cells were seeded at a density of 10,000 cells per well in 96‐well plates designed for the instrument and incubated until adhesion at 37°C overnight. The cells were then treated with the following test compounds for 24 h: 0.1% DMSO, 200 μM AICAR, 20 μM compound C, 25 μM etomoxir and 200 μM AICAR+25 μM etomoxir. The growth medium was replaced with the assay medium 1 h prior to measurement, and the cells were kept at 37°C in an atmosphere without CO_2_. The extracellular acidification rate (ECAR), an indicator of aerobic glycolysis, and the oxygen consumption rate (OCR), an indicator of oxidative phosphorylation, were measured using a Seahorse XF Glycolic Rate Assay Kit (Agilent Technologies) following the manufacturer's instructions. The glycolic proton efflux rate of HTR8/SVneo cells was measured in the presence of 0.5 μM rotenone/antimycin A and 50 mM 2‐DG.

### 2.22 Immunofluorescence

Mouse uteroplacental units were collected at GD 18.5, fixed in 4% paraformaldehyde (Bioservice, China) overnight, and then transferred to 30% sucrose. The tissues were embedded in Tissue‐Tek O.C.T. compound (Sakura) and frozen in liquid nitrogen. Ten‐micrometre‐thick cryosections were washed with PBS, permeabilized with 0.5% Triton X‐100 (Sigma–Aldrich), and then blocked with 5% bovine serum albumin (BSA; Servicebio, China). The slides were incubated with a primary mouse anti‐cytokeratin 7 polyclonal antibody (1:100; Servicebio, China) overnight at 4°C and then with fluorescein isothiocyanate (FITC)‐conjugated goat anti‐mouse IgG (1:1000, Proteintech, China) at 37°C for 1 h. The slides were washed three times with PBS after each step. The stained sections were mounted by using mounting medium containing DAPI (Vector Laboratories) and examined using a fluorescence microscope (Life Technologies) and/or confocal microscope (Zeiss, Germany).

The HTR8/SVneo cells were seeded onto coverslips, cultured until reaching 60%–70% confluence, and then fixed with 4% paraformaldehyde (Bioservice, China) for 10 min at 37°C. After washing three times in PBS, the cells were permeabilized with 0.5% Triton X‐100 (Sigma–Aldrich) and then blocked in 5% BSA (Servicebio, China) at room temperature for 1 h. Then, primary antibodies diluted in 0.1% BSA were applied, and the cells were incubated overnight at 4°C. After washing the coverslips three times with PBS, the desired fluorescent dye‐labelled secondary antibodies in 0.1% BSA were added, and the cells were incubated for 60 min at room temperature for 1 h and then washed three times in PBS. The stained cells were mounted using mounting medium containing DAPI (Vector Laboratories) and examined using a fluorescence microscope (Life Technologies) and/or confocal microscope (Zeiss, Germany).

### 2.23 Membrane protein isolation

For the isolation of membrane proteins, a Minute™ Plasma Membrane Protein Isolation Kit (Invent Biotechnologies) was used according to the manufacturer's instructions. The isolated membrane proteins were then dissolved in denaturing protein solubilization reagent (Invent Biotechnologies).

### 2.24 Immunoblotting

Proteins were extracted from placental tissues and cells with RIPA lysis buffer (Pierce) containing EDTA‐free protease inhibitor cocktail (Roche, Germany). The protein concentration was measured using a BCA Protein Assay Kit (Beyotime, China). The protein samples were loaded onto SDS‐polyacrylamide gels, electrophoresed, and transferred to polyvinylidene difluoride (PVDF) membranes (Roche, Germany). The membranes were blocked for 1 h with 5% nonfat dried milk in Tris‐buffered saline containing 0.05% Tween‐20 (TBS‐T) and then incubated with primary antibodies.

The following antibodies were purchased from Abcam (Cambridge): anti‐GLUT1 (1:1000, ab115730), anti‐Bcl2 (1:1000, ab32124), anti‐Bax (1:1000, ab32503), anti‐CAMKK2 (1:1000, ab168818), anti‐tubulin (1:1000, ab176560), anti‐Caspase9 (1:1000, ab202068) and anti‐Na^+^/K^+^ ATPase (1:1000, ab76020). The following antibodies were purchased from Cell Signalling Technology (Danvers): anti‐AMPK (1:1000, #2532), anti‐p‐AMPK (1:1000, #2535), anti‐p‐AKT (1:1000, #4060), anti‐ACC (1:1000, #3662), anti‐p‐ACC (1:1000, #3661), anti‐AKT (1:1000, #9272), anti‐cleaved Caspase3 (1:1000, #9664T), anti‐Caspase3 (1:1000, #9662 S), anti‐cleaved Caspase9 (1:1000, #9509), anti‐LKB1 (1:1000, #3047), anti‐p‐LKB1 (1:1000, #3482), anti‐IDH (1:1000, #56439) and anti‐β‐actin (1:1000, #3700). Anti‐GLUT3 (1:1000, sc‐30107), anti‐GLUT4 (1:1000, sc‐7938) and anti‐PKM (1:1000, sc‐365684) were purchased from Santa Cruz Biotechnology (Dallas). Anti‐HK2 (1:1000, #bs‐3993R) was purchased from Bioss (Beijing, China). Anti‐PFK (1:1000, #55028‐1), anti‐PDH E1α (1:1000, #18068‐1‐AP) and anti‐LDH (1:1000, #19987‐1‐AP) were purchased from Proteintech (Chicago). Anti‐p‐p53 (1:1000, GTX634168) and anti‐p53 (1:1000, GTX70214) were purchased from Genetex (San Diego).

The membranes were washed and incubated with secondary antibodies linked to horseradish peroxidase (Santa Cruz Biotechnology). Reagents for Western blot detection by enhanced chemiluminescence (ECL) were purchased from Millipore (Billerica), and images were captured using a ChemiDoc (Bio‐Rad). The density of bands was analysed by Bio‐Rad Image Lab Software (Bio‐Rad).

### 2.25 RT‐qPCR

RNA extraction and cDNA reverse transcription were performed as previously described.^28^ Cell RNA was isolated with a total RNA isolation kit (Bioteke, China) according to the manufacturer's instructions. The primers were synthesized by Takara (Takara, Japan), and the sequences of the primers are listed in Table S3. The mRNA levels were quantified using SYBR Green (Roche, Germany), and melt curve analysis was performed to ensure amplification specificity. Forty cycles of PCR were performed in a Bio‐Rad CFX Connect™ Real‐Time System (Bio‐Rad). The initial enzyme activation and template denaturation at 95°C for 10 min were followed by denaturation at 95°C for 5 s, annealing at 63.3°C for 30 s and extension at 72°C for 10 s, after which melt curve analysis was performed. Cycle threshold (Ct) values were used for quantification. The efficiency values for all assays were 95%–105%.

### 2.26 Flow cytometry

Apoptosis was assessed by flow cytometry as previously established by our group.^29^ Briefly, cells (>1 × 10^6^) were harvested after treatment by centrifugation at 1000 rpm for 5 min and resuspended in 500 μl of PBS. Then, the cells were analysed using a FACScan flow cytometer (CytoFLEX, China) after staining with Annexin V‐FITC (in the blank group) or Annexin V‐PE‐A (in the sh*SLC2A3* and shNC groups).

### 2.27 Lactic acid and pH measurement

When the cells grew to 80%–90% confluence, the cells and cell culture medium were harvested separately. Cell lysates were extracted with RIPA lysis buffer (Pierce) containing EDTA‐free protease inhibitor cocktail (Roche, Germany). The pH and lactic acid levels in the cell culture medium and cell lysate were then determined by a haematology analyser (GEM).

### 2.28 Transwell assay

Matrigel‐coated Transwell assays were performed as previously reported.^30^ HTR8/SVneo cells (8 × 10^4^) were plated in transwell inserts (8.0 μm, Merck Millipore, Germany) precoated with 60 μl of Matrigel (BD Biosciences). After 4 h of incubation at 37°C, 200 μl of RPMI Medium 1640 basic (1×; Life Technologies) containing vehicle (0.1% DMSO), AICAR (Selleck), compound C (Selleck) or PEP was loaded into the upper chambers, while 500 μl of medium containing 10% FBS was added into the lower chambers. After incubation in 21% O_2_ or 1% O_2_ at 37°C for 24 h, the noninvading cells on the top of the inserts were scraped off using a cotton swab. The filter under the inserts with invaded cells attached was washed with cold PBS, and the cells were fixed with 4% paraformaldehyde, stained with crystal violet and photographed with an EVOS FL Auto microscope (Life Technologies). In each independent experiment, a scan of the entire field of view was obtained for a comprehensive description of each sample, and 10 fields of view at 200× magnification were randomly selected per sample for quantification.

### 2.29 Cell migration assay

The migration of HTR8/SVneo cells was assessed by wound healing tests as previously described.^31^ Briefly, a scratch lesion was created on confluent trophoblast cultures with a 200‐μl pipette tip, and photographs were captured immediately and 15 h later by an EVOS FL Auto microscope (Life Technologies). Three fields of view at the lesion border were randomly selected. The images were then analysed using ImageJ software (National Institutes of Health).

### 2.30 Gelatin zymography

Gelatin zymography was performed as previously described.^31^, ^32^ The cell culture medium was centrifuged at 13,000*g* for 15 min, and the supernatant was then collected for electrophoresis. Following gel electrophoresis, the gels were removed, washed three times with TBS‐T for 30 min at room temperature on a shaking platform and then incubated in digestive solution (3.03 g of Tris, 4.4 g of NaCl, 0.55 g of CaCl_3_ and 0.1 g of NaN_3_ in a total volume of 500 ml with an adjusted pH of 7.6) at room temperature for 30 min. The digestive solution was replaced, and the gels were incubated for 48 h at 37°C on a shaking platform at 50 rpm. The gels were then stained in staining buffer (2.0 g of Coomassie blue, 100 ml of isopropyl alcohol and 40 ml of glacial acetic acid in a total volume of 400 ml) for 1 h on a shaking table at 50 rpm at room temperature and destained in destaining buffer consisting of methanol, glacial acetic acid and double‐distilled water. Finally, the gels were scanned by a Quantity One System image analyser (Bio‐Rad).

### 2.31 Statistical analysis

Statistical analysis was performed using Prism 6 (GraphPad). The bars in the graphs indicate the means ± SEMs. Comparisons between two groups were carried out by a two‐tailed Student's *t*‐test. Comparisons among multiple groups were carried out by one‐way analysis of variance (ANOVA) followed by Tukey's multiple comparisons test. Two‐way ANOVA followed by Tukey's multiple comparison test was used to analyse data from multiple groups with multiple characteristics. For all analyses, a *p*‐value <0.05 was considered to indicate significance.

## 3 RESULTS

### 3.1 PE is associated with placental AMPK hyperactivation

**FIGURE 1:**
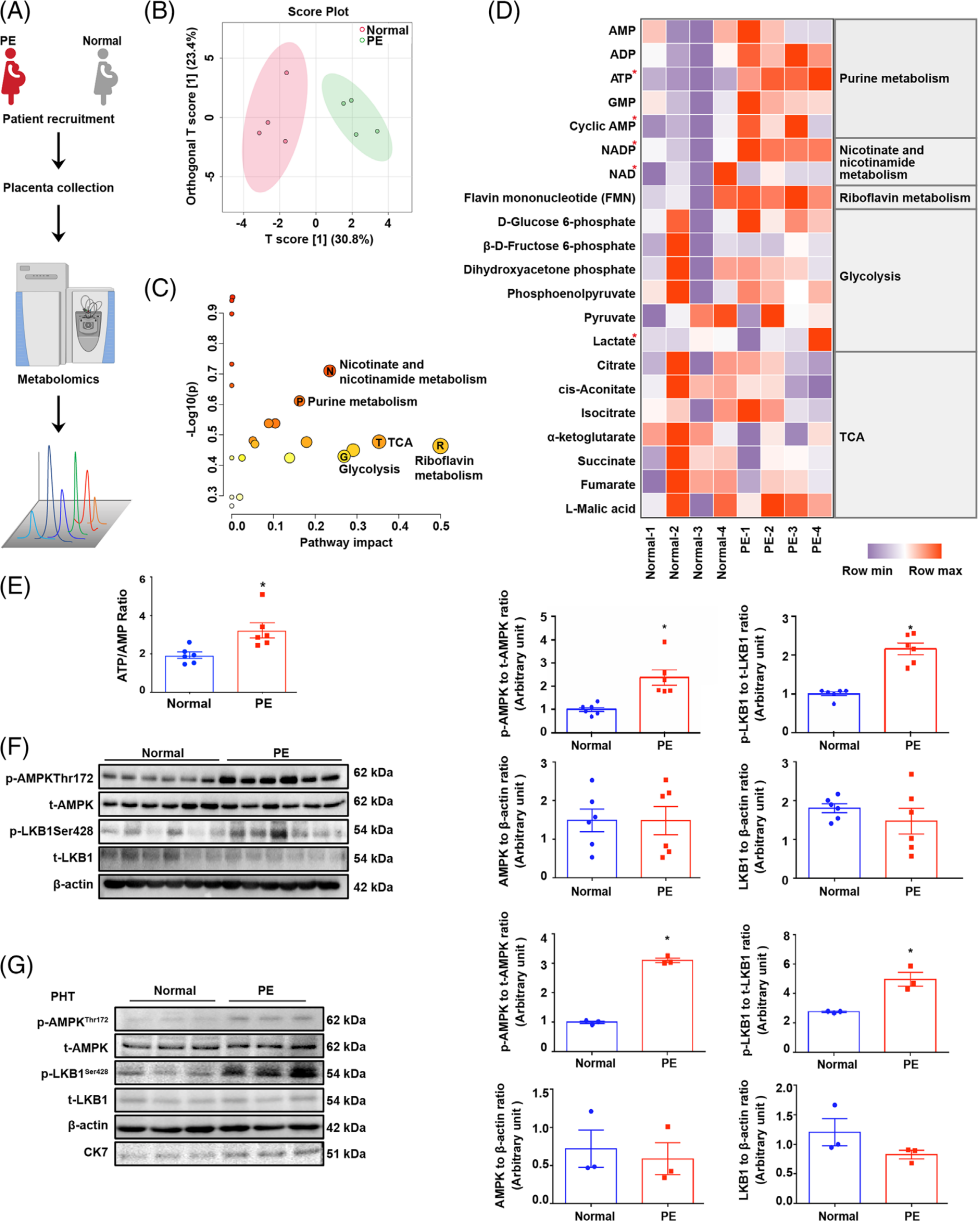
PE is associated with elevated phosphorylation of AMPK in the placenta. (A) Schematic representation of the experimental design. Control and PE term placentas were collected for metabolomics profiling. (B) The OPLS‐DA results show that discrimination between the control group and PE patients was optimized. (C) Pathway analysis of metabolites in control and PE placentas using MetaboAnalyst 4.0. The metabolomics overview shows all the matched pathways as circles arranged according to the scores from enrichment analysis (*y* axis) and topology analysis (*x* axis). The colour and size of each circle are based on the P and pathway impact values, respectively. (D) The heatmap shows the energy metabolism intermediates in human term placentas. (E) The ATP/AMP ratio was quantified by commercially available kits. *n* = 6–13, two‐tailed *t*‐test, *p < 0.05. (F) Western blots of p‐AMPK, t‐AMPK, p‐LKB1 and t‐LKB1 in human term placentas. *n* = 6, two‐tailed *t*‐test, *p < 0.05. (G) Western blots of p‐AMPK, t‐AMPK, p‐LKB1, t‐LKB1 and CK7 in PHTs isolated from term placentas. *n* = 3, two‐tailed *t*‐test, *p < 0.05. All data are presented as the mean ± SEM

Although PE has long been assumed to be an ischemic placental disease, its metabolic features have not yet been fully described. Therefore, in this study, we analysed energy metabolism in the placentas of untreated PE patients by gas chromatography–mass spectrometry (GC–MS) for the first time (Figure 1A). According to the orthogonal partial least‐squares‐discriminant analysis (OPLS‐DA) model, metabolites from the control group and PE groups showed optimal discrimination ability (R2Xcum = 0.308, R2Ycum = 0.85, Q2cum = 0.673; Figure 1B). Intriguingly, the ATP content in PE‐complicated placentas was significantly higher than that in normal placentas (Figure 1C,D). To validate these findings from metabolomic analysis, we next quantified the ATP/AMP ratio by commercially available assay kits (Figure 1E). Eukaryotes have evolved a very sophisticated system for metabolic modulation. The key guardian in this system is AMPK, which is responsible for ATP production.^33^ Previous finding of our laboratory that hyperphosphorylation of AMPK in PE placentas^34^ was validated in this study. Most importantly, we observed that the phosphorylation of LKB1, the upstream kinase of AMPK, was also greater in PE placentas than in normal placentas (Figure 1F). Moreover, this activation of the LKB1‐AMPK axis was confirmed in primary human trophoblasts (PHTs) isolated from PE placentas (Figure 1G and Figure S1).

### 3.2 Activation of trophoblastic AMPK during placentation exacerbates PE

**FIGURE 2:**
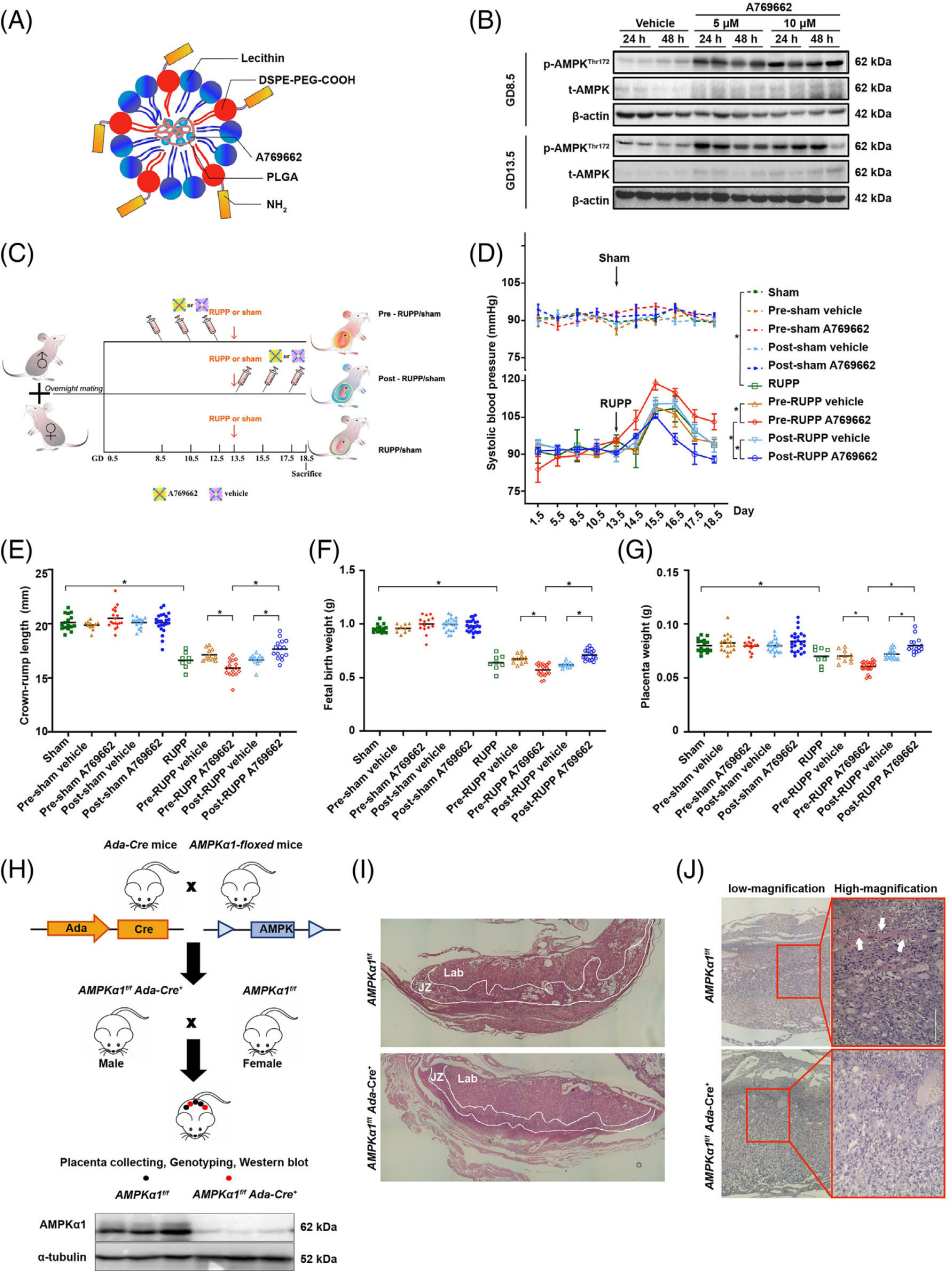
Activation of trophoblast AMPK during placentation exacerbates the PE phenotype in a mouse RUPP model. (A) Schematic illustration of the composition and structure of plCSA‐NPs. (B) Mouse placental AMPKα1 activation after treatment with A769662‐loaded plCSA‐NPs on GD 8.5 or GD 13.5. (C) Schematic of the treatment plan. (D) Systolic blood pressure. *n* = 6 animals; two‐way ANOVA and Tukey's multiple comparison test; *p < 0.05. (E) Fetal crown‐rump length. *n* = 8–25 pups from three dams; one‐way ANOVA and Tukey's multiple comparison test; *p < 0.05. (F) Fetal birth weight. *n* = 8–25 pups from three dams; one‐way ANOVA and Tukey's multiple comparison test; *p < 0.05. (G) Placental weight. *n* = 8–25 pups from three dams; one‐way ANOVA and Tukey's multiple comparison test; *p < 0.05. (H) Schematic representation of the mating strategy used to generate AMPK trophoblast‐specific knockout strains. (I) H&E staining and (J) PAS staining of placental sections from *AMPKα1* ^*f/f*^ and *AMPKα1* ^*f/f*^ *Ada*‐Cre^+^ mice; the white arrows indicate positive staining. All data are presented as the mean ± SEM

To explore whether placental AMPK hyperactivation is the driving force in the development of PE, we first established an RUPP‐induced mouse PE model (Figure S2A), which demonstrated compromised uterine artery flow velocities (Figure S2B) and morphological changes in the kidney (Figure S2C). Then, placenta‐specific AMPK activation was achieved by tail vein injection of plCSA‐NPs loaded with the AMPK‐specific agonist A769662^35^ (plCSA‐A769662) (Figure 2A). The specificity of delivery to the placenta in pregnant mice was confirmed by in vivo imaging (Figure S2D). Various doses of plCSA‐A769662 were administered to pregnant mice in early or late gestation, and 5 μM A769662 delivered by plCSA‐NPs effectively activated placental AMPK for at least 24 h (Figure 2B) without affecting AMPK in the brain, heart, kidneys or liver (Figure S2E–H). Therefore, 5 μM plCSA‐A769662 or control plCSA‐NPs were then administered to RUPP or sham mice either before or after the operation (GD 13.5), as illustrated in Figure 2C. Pre‐RUPP administration of plCSA‐A769662 led to a significant increase in the average post‐surgery systolic pressure for RUPP mice (108.9 ± 5.6 mmHg vs. 99.9 ± 0.1 mmHg in the pre‐RUPP vehicle group), while post‐RUPP plCSA‐A769662 treatment ameliorated RUPP‐induced hypertension (96.1 ± 1.4 mmHg vs. 102.0 ± 1.6 mmHg in the post‐RUPP vehicle group; Figure 2D). Accordingly, although RUPP significantly augmented serum sFlt‐1 levels and urinary albumin levels, the levels of these molecules were further enhanced by pre‐RUPP plCSA‐A769662 administration from GD 8.5 (Figure S3A,B). In accordance with this finding, pre‐surgery administration of plCSA‐A769662 deteriorated RUPP‐induced kidney injury (Figure S3C–E). Moreover, activation of placental AMPK during placentation exacerbated RUPP‐related reductions in crown‐rump length, fetal birth weight and placental weight, all of which were rescued when plCSA‐A769662 was administered on GD 13.5 (Figure 2E–G). In contrast, activation of placental AMPK during late pregnancy relieved RUPP‐induced sFlt‐1 elevations, proteinuria, kidney injury and fetal growth restriction.

### 3.3 Trophoblastic AMPK dysregulation disturbs placental structure in mice

Then, to study the effects of AMPK activation alone on pregnancy outcome, plCSA‐A769662 and vehicle were administered on GD 8.5 and GD 13.5, and placental labyrinth development was assessed on GD 18.5. The results indicated that placental AMPK activation in early gestation significantly reduced the labyrinth zone (LZ)/junctional zone (JZ) thickness ratio, while AMPK activation in late gestation had no effect on placental development (Figure S4A). Administration of plCSA‐A769662 in late gestation reduced placental TUNEL‐positive signals, in accordance with the protective effect against RUPP‐induced PE manifestation (Figure S4B). In contrast, increased placental TUNEL‐positive signals were observed in term placentas of the early gestation plCSA‐A769662 treatment group, probably due to maldevelopment of the labyrinth and consequent hypoperfusion in the placenta.

Furthermore, the establishment of trophoblast‐specific *AMPKα1‐*deletion (*AMPKα1* ^*f/f*^ *Ada*‐Cre^+^) mice provided opportunities to elucidate the functional role of AMPK in placental development (Figure 2H). AMPK protein levels in the placenta on GD 18.5 were detected by Western blot analysis (Figure 2H). The JZ of the mouse placenta consists of two main trophoblast populations, spongiotrophoblasts (spTs) and glycogen cells (glyTs). Between GD 12.5 and GD 16.5, the number of glyTs increases by more than 250‐fold,^36^ representing a significant increase in the storage of glucose for potential use by the embryo in late gestation. On GD 18.5, glyTs are characterized by their vacuolated cytoplasm, and there are 40% fewer glyTs at GD 18.5 than at GD 16.5.^36^ Our results showed that in *AMPKα1* ^*f/f*^ *Ada*‐Cre^+^ placentas, a maximally altered placental structure was characterized by dramatically reduced numbers of glyTs in the JZ (Figure 2I) and a lack of glycogen deposits (Figure 2J), which are related to impaired fetal growth.^37^ Thus, either hyperactivation or loss of trophoblastic AMPK during placentation interferes with placental development, possibly by disrupting glucose metabolism, which indicates that placental AMPK must be precisely modulated to ensure successful perinatal outcomes.

### 3.4 AMPK activation suppresses trophoblast invasion while preserving trophoblast viability

**FIGURE 3:**
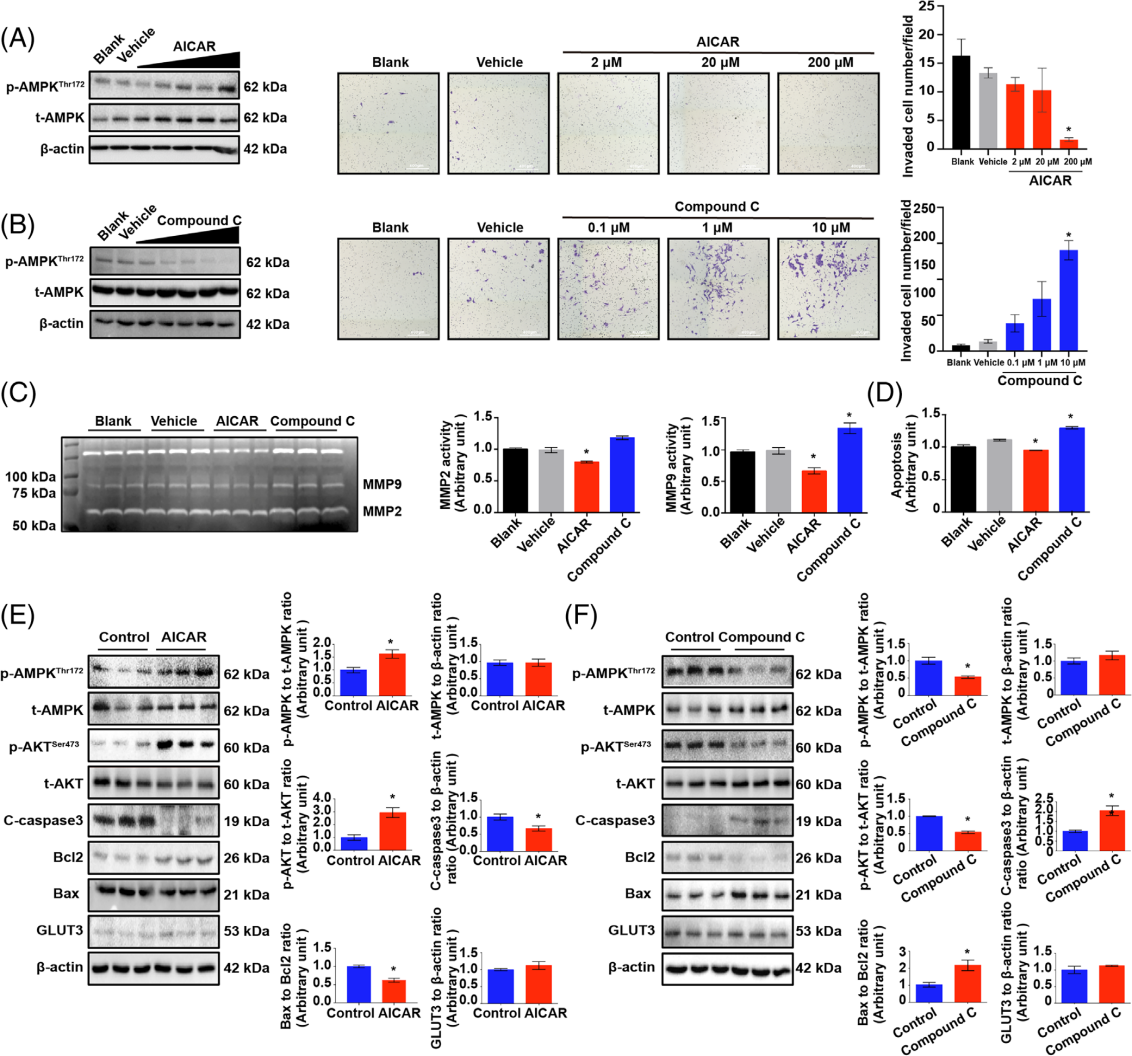
AMPK regulates homeostasis between trophoblast invasion and viability. (A, B) HTR8/SVneo cells were treated with various doses of AICAR (A) and compound C (B), and the invasion of HTR8/SVneo cells was monitored via Transwell assays in the presence of different doses of AICAR. The blank and vehicle (0.1% DMSO) groups were included as controls. *n* = 3; one‐way ANOVA and Tukey's multiple comparison test; *p < 0.05 vs. vehicle (0.1% DMSO). The p‐AMPK levels were determined by Western blotting. (C) Gelatin zymography of MMP‐2 and MMP‐9 in the culture medium of HTR8/SVneo cells treated with 200 μM AICAR or 10 μM compound C for 24 h. *n* = 3; one‐way ANOVA and Tukey's multiple comparison test; *p < 0.05 versus vehicle (0.1% DMSO). (D) Apoptosis of HTR8/SVneo cells after 24 h of incubation with 200 μM AICAR or 10 μM compound C as measured by flow cytometry. *n* = 3; one‐way ANOVA and Tukey's multiple comparison test; *p < 0.05 versus vehicle (0.1% DMSO). (E) Western blots of p‐AMPK, t‐AMPK, p‐AKT, t‐AKT, C‐caspase3, Bcl2, Bax and GLUT3 in HTR8/SVneo cells treated with 200 μM AICAR for 24 h. *n* = 3; two‐tailed *t*‐test; *p < 0.05. (F) Western blots of p‐AMPK, t‐AMPK, p‐AKT, t‐AKT, C‐caspase3, Bcl2, Bax and GLUT3 in HTR8/SVneo cells treated with 10 μM compound C for 24 h. *n* = 3; two‐tailed *t*‐test; *p < 0.05. All data are presented as the mean ± SEM

**FIGURE 4:**
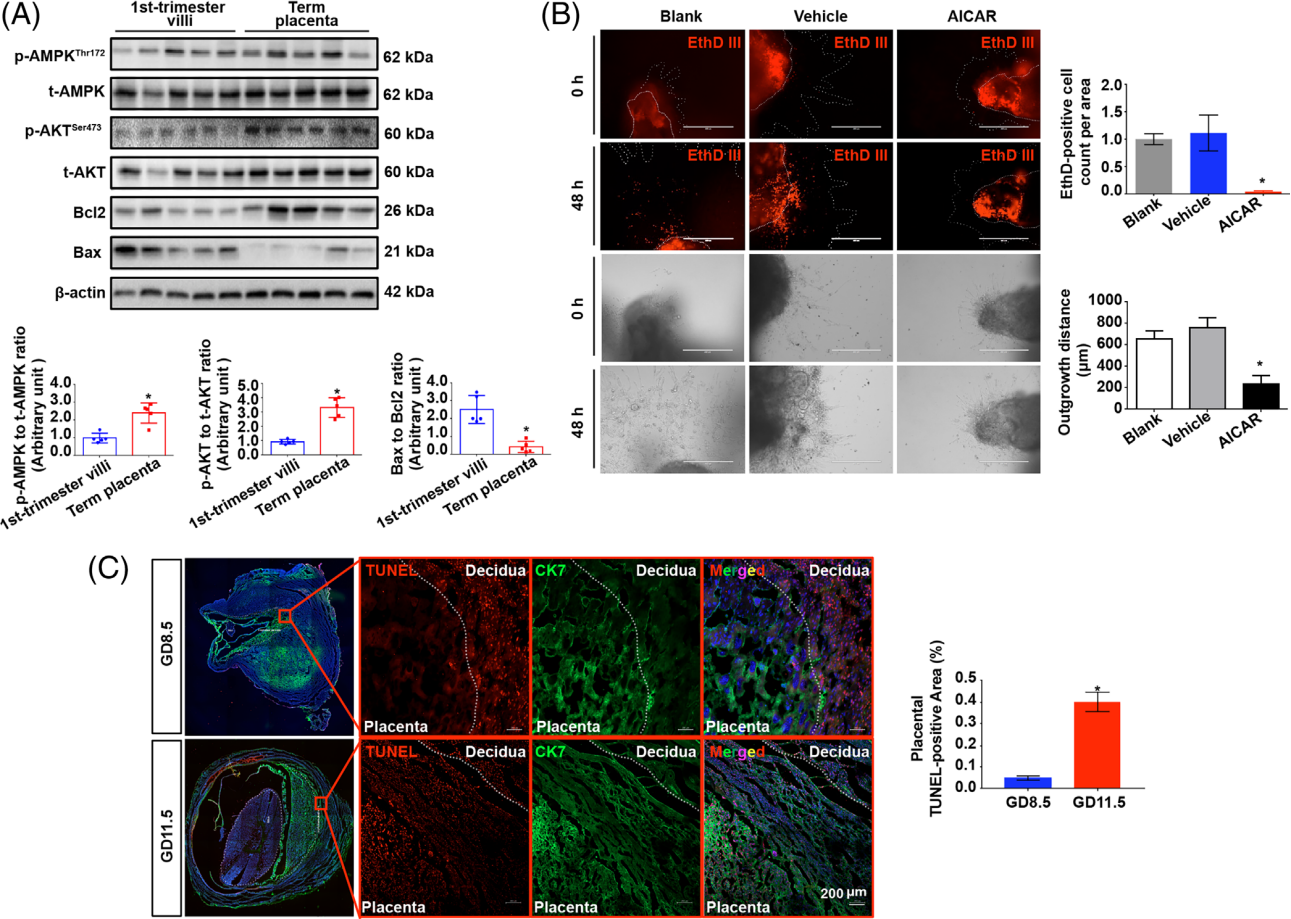
In the late gestational stage, trophoblasts exhibit higher viability in both humans and mice. (A) Western blots of p‐AMPK, t‐AMPK, p‐AKT, t‐AKT, Bcl2 and Bax in first‐trimester villi and term placentas from uncomplicated human pregnancies. *n* = 5; two‐tailed *t*‐test; *p < 0.05. (B) Villus outgrowth after treatment with 200 μM AICAR for 48 h. Cell death was detected with EthD III. *n* = 3; one‐way ANOVA and Tukey's multiple comparison test; *p < 0.05. (C) TUNEL staining of mouse placentas collected on GD 8.5 or GD 13.5. Scale bar 200 μm; *n* = 3; two‐tailed *t*‐test; *p < 0.05. All data are presented as the mean ± SEM

To further confirm whether AMPK hyperphosphorylation‐associated labyrinth maldevelopment is due to defective trophoblast invasion, HTR8/SVneo trophoblasts were treated with various doses of the specific AMPK agonist AICAR.^38^, ^39^ The invasion of treated cells gradually decreased with increasing concentrations of AICAR (Figure 3A). In a reciprocal experiment, different doses of the AMPK antagonist compound C^40^ were applied. The invasion of trophoblasts was promoted by compound C in a dose‐dependent manner (Figure 3B). In accordance with the results of the invasion assay, we also found that AICAR suppressed the activity of MMP2 and MMP9, while compound C exhibited the opposite effects (Figure 3C). Taken together, our findings suggest that AMPK hyperactivation is associated with the pathological mechanism of PE via negative regulation of trophoblast invasion. Nevertheless, given that unexpected cell death may have resulted in the reduction in cell invasion, the effects of AICAR and compound C on the survival of HTR8/SVneo cells were examined by flow cytometry. The results showed that AICAR treatment suppressed apoptosis in trophoblasts, while compound C significantly elevated apoptosis (Figure 3D). Consistent with these results, AICAR significantly elevated the phosphorylation of AKT but dramatically suppressed the Bax/Bcl2 ratio and the levels of cleaved‐caspase3 (Figure 3E). In contrast, compound C substantially inhibited the phosphorylation of both AMPK and AKT but increased the Bax/Bcl2 ratio and cleavage of caspase3 (Figure 3F). To confirm the homeostasis of invasiveness and viability during different periods of physiological pregnancy, cell death and survival signals in first‐trimester human villi, which contain more aggressive extravillous trophoblasts (EVTs) than term placentas, and term placentas that had lost invasive trophoblasts were assessed. The results indicated that the Bax/Bcl2 ratio was significantly higher in villi than in term placentas, while villi lacked p‐AMPK and p‐AKT (Figure 4A). In addition, EthD III‐stained cells exhibited increased villous sprouting areas, whereas AICAR treatment markedly rescued cell death and resulted in significant suppression of villous outgrowth (Figure 4B). Furthermore, apoptosis was barely detected in mouse placentas on GD 8.5, at which time the trophoblasts had just begun to acquire invasiveness; in contrast, the number of apoptotic cells was dramatically increased on GD 11.5, when the trophoblasts had gained maximal invasiveness to expand into the placentas (Figure 4C).

### 3.5 AMPK activation induces glucose uptake by enhancing trophoblastic GLUT3 translocation to the plasma membrane

**FIGURE 5:**
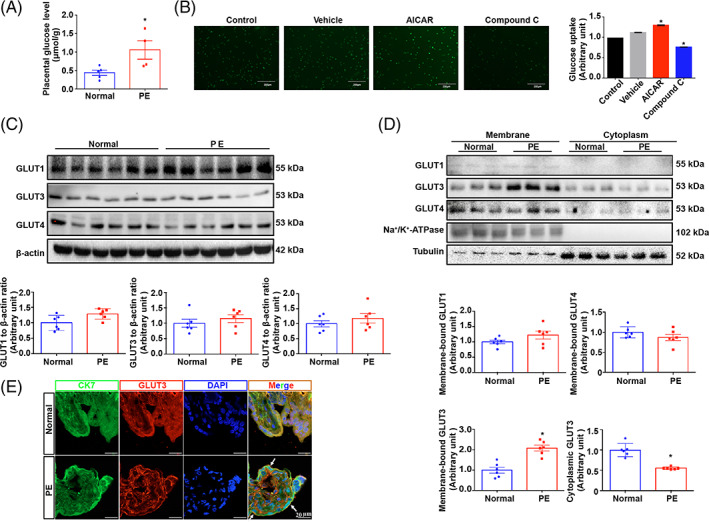
Trophoblast GLUT3 cytosol‐plasma translocation is identified in the PE placenta. (A) Glucose levels of control and PE placentas. *n* = 4; two‐tailed *t*‐test; *p < 0.05. (B) 2‐NBDG uptake into HTR8/SVneo cells after 24 h of treatment with 200 μM AICAR or 10 μM compound C. NC (2‐NBDG‐free), control and vehicle (0.1% DMSO)‐treated cells were included. *n* = 3; one‐way ANOVA and Tukey's multiple comparison test; *p < 0.05 versus vehicle. (C) Expression levels of GLUT1, GLUT3 and GLUT4 in human term placentas; β‐actin was used as a loading control. *n* = 6; two‐tailed *t*‐test. (D) The membrane‐bound GLUT and cytoplasmic GLUT expression levels in human term placentas. Tubulin and Na^+^/K^+^ ATPase were blotted as loading controls. *n* = 6; two‐tailed *t*‐test; *p < 0.05. (E) IF staining of GLUT3 in normal and PE‐complicated human term placentas. Scale bar 20 μm. All data are presented as the mean ± SEM

**FIGURE 6:**
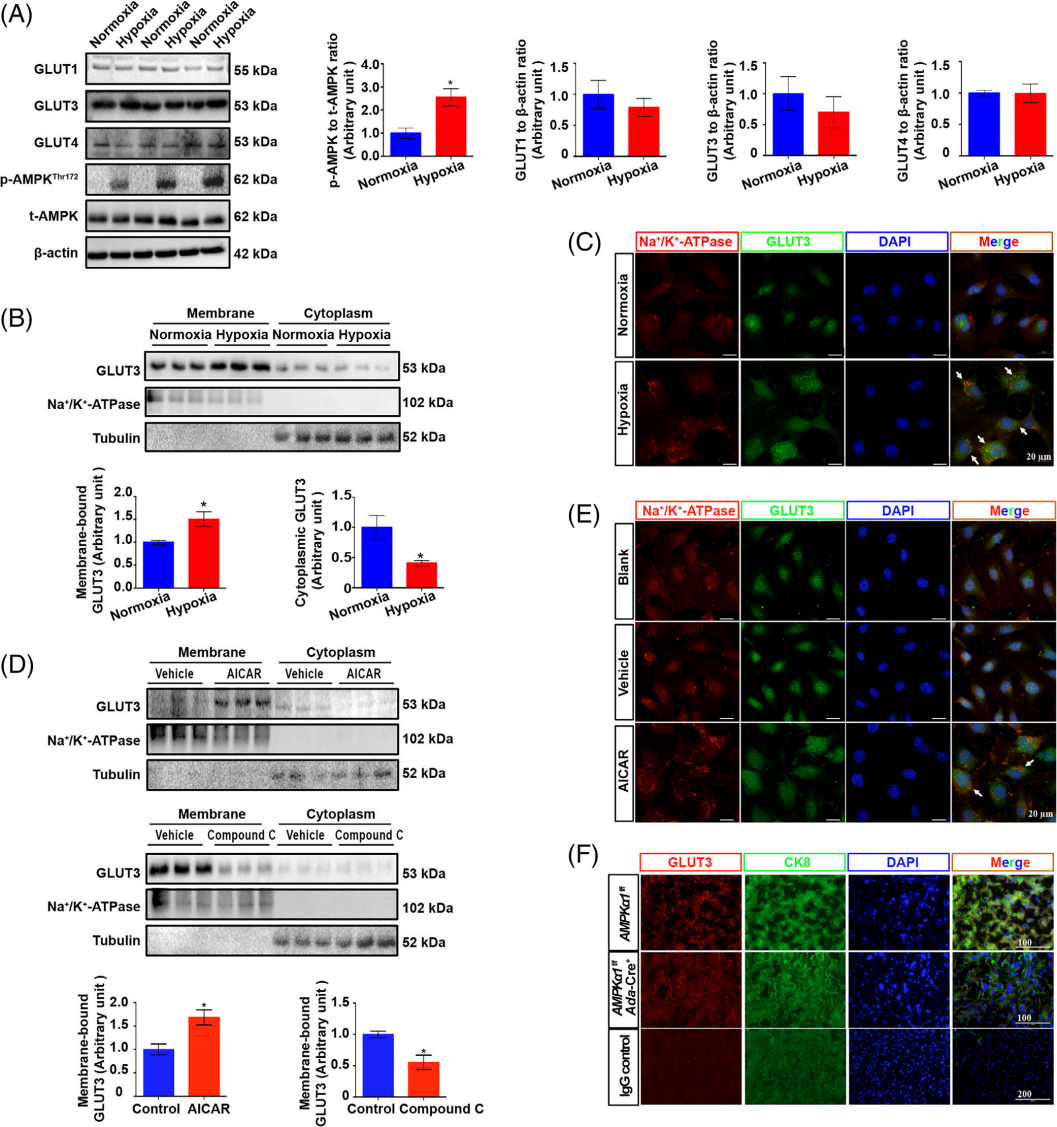
Trophoblast GLUT3 cytosol‐plasma translocation is modulated by AMPK activity. (A) HTR8/SVneo cells were subjected to hypoxia (1% O_2_) and normoxia (21% O_2_) for 24 h, harvested for immunoblotting and incubated with antibodies against GLUT1, GLUT3, GLUT4, t‐AMPK and p‐AMPK. *n* = 3; two‐tailed *t*‐test; *p < 0.05. (B) The membrane‐bound and cytoplasmic GLUT3 protein levels. Tubulin and Na^+^/K^+^ ATPase were used as loading controls. *n* = 3; *p < 0.05. (C) IF staining of GLUT3. Scale bar 20 μm. (D) HTR8/SVneo cells were treated with 200 μM AICAR or 10 μM compound C for 24 h, and the plasma membrane and cytoplasmic fractions were then separated for immunoblotting of GLUT3. *n* = 3; two‐tailed *t*‐test; *p < 0.05. Tubulin and Na^+^/K^+^ ATPase were blotted as loading controls in both experiments. (E) IF staining of GLUT3 in HTR8/SVneo cells after 24 h of 200 μM AICAR treatment. Scale bar 20 μm. (F) IF staining of placental sections from *AMPKα1* ^*f/f*^ and *AMPKα1* ^*f/f*^ *Ada*‐Cre^+^ mice. All data are presented as the mean ± SEM

To study whether activation of AMPK enhances glucose uptake in trophoblasts, we examined the glucose content in humans. Unsurprisingly, the levels of glucose were significantly higher in human PE placentas than in controls (Figure 5A). A 2‐NBDG uptake assay further confirmed that AICAR treatment significantly enhanced glucose uptake into HTR8/SVneo cells, while compound C largely alleviated glucose uptake (Figure 5B). To further evaluate the contribution of GLUT3 to glucose transport into trophoblasts, 2‐deoxy‐d‐[^3^H] glucose uptake was analysed, and the results showed that a reduction in GLUT3 expression by nearly half resulted in a significant reduction in glucose uptake that was comparable to the effect of GLUT1 inhibition with fasentin and was half as strong as the effect of blocking both GLUT1 and GLUT4 with WZB117 (Figure S5H). These findings indicate that GLUT3 plays a role equivalent to that of GLUT1 in glucose uptake in trophoblasts. Moreover, the total GLUT1, GLUT3 and GLUT4 protein expression levels did not differ between PE and normal placentas (Figure 5C). Intriguingly, although the membrane localization of neither GLUT1 nor GLUT4 was altered in PE placentas, the level of cytoplasmic membrane‐bound GLUT3 was significantly elevated in PE placentas, and those of cytoplasmic GLUT3 were accordingly reduced in PE placentas (Figure 5D,E). To ascertain whether enhanced GLUT3 trafficking from the cytosol to the plasma membrane in PE placentas is a result of AMPK hyperactivation, an in vitro HTR8/SVneo trophoblastic PE model was established via hypoxia as described previously.^41^ The results showed that compared with normoxia, hypoxia significantly promoted the phosphorylation of AMPK in trophoblasts without modifying the total expression of GLUTs (Figure 6A). Importantly, the cytoplasmic membrane‐bound GLUT3 level was increased by 50.5%, in accordance with a 59.2% reduction in the cytoplasmic GLUT3 level (Figure 6B). The redistribution of GLUT3 in HTR8/SVneo cells induced by hypoxia was further visualized by IF staining (Figure 6C). Specifically, AMPK activity in HTR8/SVneo cells was modulated by AICAR or compound C treatment. The data clearly showed that the translocation of GLUT3 from the cytoplasm to the plasma membrane was promoted by AICAR but inhibited by compound C (Figure 6D,E). In addition, *AMPK1α* ^*f/f*^ *ADA‐Cre* ^+^ placentas exhibited lower levels of membranous GLUT3 than WT control placentas, indicating that trophoblastic GLUT3 translocation onto the plasma membrane is regulated by AMPK (Figure 6F).

### 3.6 GLUT3 mediates the inhibitory effects of AMPK on trophoblast invasion and migration

**FIGURE 7:**
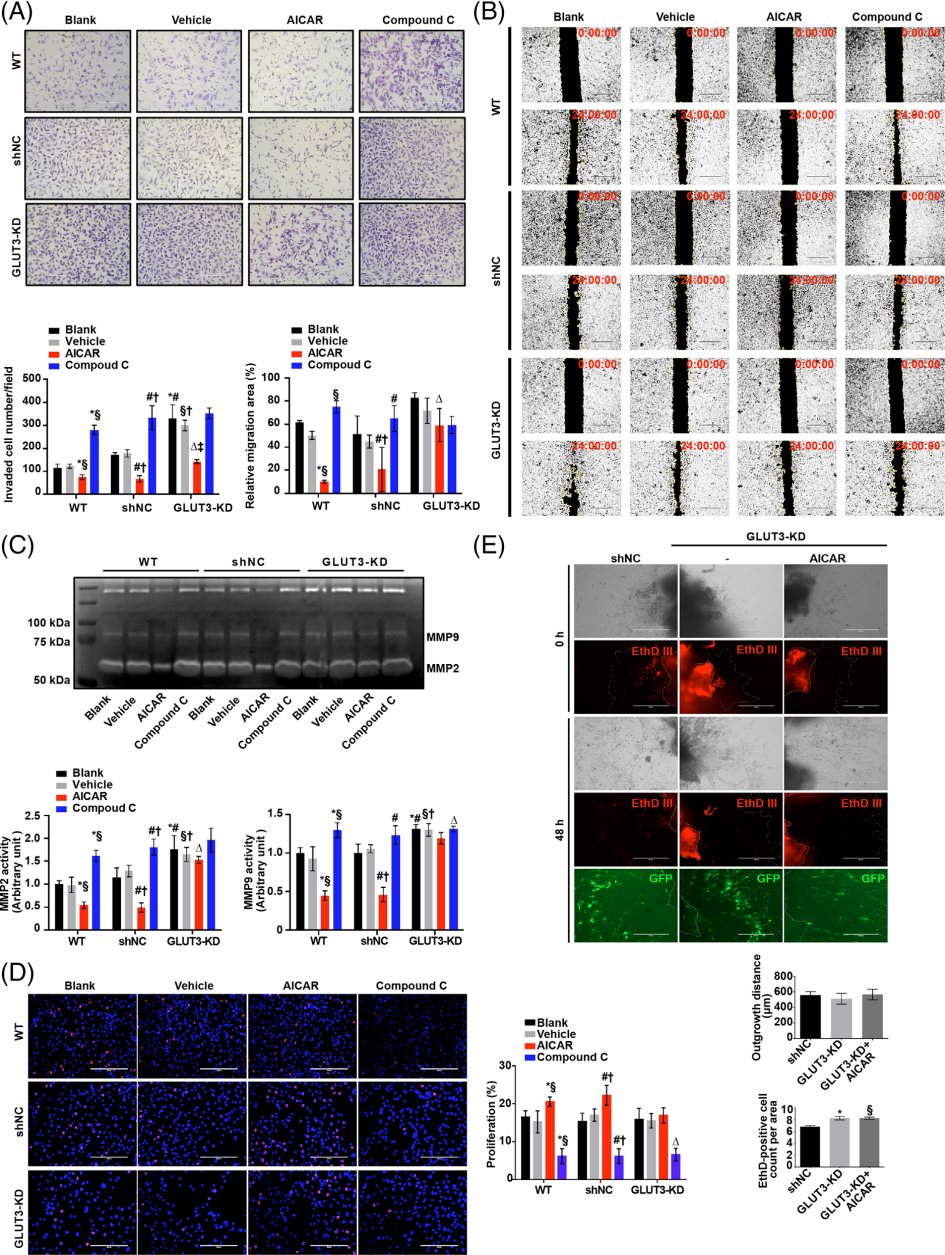
Downregulation of GLUT3 abolishes the regulatory effects of AMPK on trophoblast invasion and viability. (A) Untransfected and shNC‐ or sh*SLC2A3*‐transfected HTR8/SVneo cells were subjected to Matrigel Transwell assays in the presence of vehicle (0.1% DMSO), 200 μM AICAR or 10 μM compound C. Invaded cells were stained and counted after 24 h of treatment. Blank controls were included. *n* = 3; two‐way ANOVA and Tukey's multiple comparison test; *p < 0.05 versus WT blank, §*p* < 0.05 versus WT vehicle, #*p* < 0.05 versus shNC blank, †*p* < 0.05 versus shNC vehicle, ∆*p* < 0.05 versus WT AICAR and shNC AICAR, ‡*p* < 0.05 versus GLUT3‐KD blank and GLUT3‐KD vehicle. (B) Wound‐healing assays for the aforementioned groups of cells in the presence of vehicle (0.1% DMSO), 200 μM AICAR or 10 μM compound C. Blank controls were included. The images were obtained after 0 and 15 h of treatment. The quantified migration areas are shown in the bar graph. *n* = 3; two‐way ANOVA and Tukey's multiple comparison test; *p < 0.05 versus WT blank, §*p* < 0.05 versus WT vehicle, #*p* < 0.05 versus shNC blank, †*p* < 0.05 versus shNC vehicle, ∆*p* < 0.05 versus WT AICAR and shNC AICAR. (C) Gelatin zymography of MMP‐2 and MMP‐9 in the culture medium of HTR8/SVneo cells treated with 200 μM AICAR or 10 μM compound C for 24 h. *n* = 3; two‐way ANOVA and Tukey's multiple comparison test; *p < 0.05 versus WT blank, §*p* < 0.05 versus WT vehicle, #*p* < 0.05 versus shNC blank, †*p* < 0.05 versus shNC vehicle, ∆p < 0.05 versus WT AICAR and shNC AICAR. (D) Representative images and quantification of EdU staining in untransfected, shNC‐transfected or GLUT3‐KD cells. *n* = 3; two‐way ANOVA and Tukey's multiple comparison test; *p < 0.05 versus WT blank, §*p* < 0.05 versus WT vehicle, #*p* < 0.05 versus shNC blank, †*p* < 0.05 versus shNC vehicle, ∆*p* < 0.05 versus GLUT3‐KD blank and GLUT3‐KD vehicle. (E) Villus outgrowth after transfection with shNC or sh*SLC2A3* followed by treatment with 200 μM AICAR for 48 h. Cell death was detected with EthD III. *n* = 3; one‐way ANOVA and Tukey's multiple comparison test; *p < 0.05 versus shNC, §*p* < 0.05 versus shNC. All data are presented as the mean ± SEM

To investigate whether GLUT3 is involved in the regulatory effects of AMPK on trophoblast invasion and viability, a GFP‐tagged GLUT3‐KD HTR8/SVneo cell line was established by transfection with shRNA targeting *SLC2A3* (Figure S5A,B). The total and membranous GLUT3 levels were nearly 50% lower in GLUT3‐KD HTR8/SVneo cells than in WT HTR8/SVneo cells (Figure S5C–E), while GLUT1, GLUT4 and phosphorylation of ACC were unaffected (Figure S5F,G). These findings indicate that a substantial amount of glucose taken up via GLUT3 may not be used for energy production in trophoblasts, as ablation of GLUT3 did not lead to adaptive upregulation of key proteins in glucose and fatty acid metabolism. AICAR treatment significantly reduced invasiveness in the WT and shNC groups, while compound C significantly enhanced invasiveness in these two groups (Figure 7A). In contrast, GLUT3‐KD cells demonstrated markedly increased invasion under basal conditions. Moreover, the stimulative effect of compound C on invasion was dramatically blunted in GLUT3‐KD cells, while the inhibitory effect of AICAR was slightly attenuated. Similarly, a wound‐healing assay showed that the AICAR‐mediated enhancement and the compound C‐mediated suppression of cell migration were both largely attenuated in GLUT3‐KD cells (Figure 7B). Consistently, gelatin zymography showed that AICAR‐mediated inhibition of MMP2/9 and compound C‐mediated activation of MMP2/9 were blocked in GLUT3‐KD cells (Figure 7C).

Then, we investigated whether GLUT3 plays a role in AMPK‐mediated regulatory effects on trophoblastic viability. The results illustrated that AICAR‐induced proliferation was observed only in WT and NC cells but not in GLUT3‐KD cells (Figure 7D). However, a compound C‐mediated reduction in proliferation was also observed in GLUT3‐KD cells, which implied that other molecules may also function in this regulatory pathway. Consistent with the increased apoptosis levels in GLUT3‐KD cells (Figure S5I), reduced p‐AKT levels and increased Bax/Bcl2 ratios were revealed by a Western blot (Figure S5J). To further determine whether GLUT3 mediated AMPK's regulatory effects on the homeostasis of trophoblast invasion and viability, sh*SLC2A3*‐transfected normal human first‐trimester villi were treated with or without AICAR. The results revealed that AICAR administration neither inhibited villus outgrowth nor attenuated cell death in the presence of sh*SLC2A3* (Figure 7E). Taken together, these findings indicate that downregulation of GLUT3 attenuates the regulatory effects of AMPK on both survival and invasion.

### 3.7 AMPK hyperactivation does not support the production of lactic acid

**FIGURE 8:**
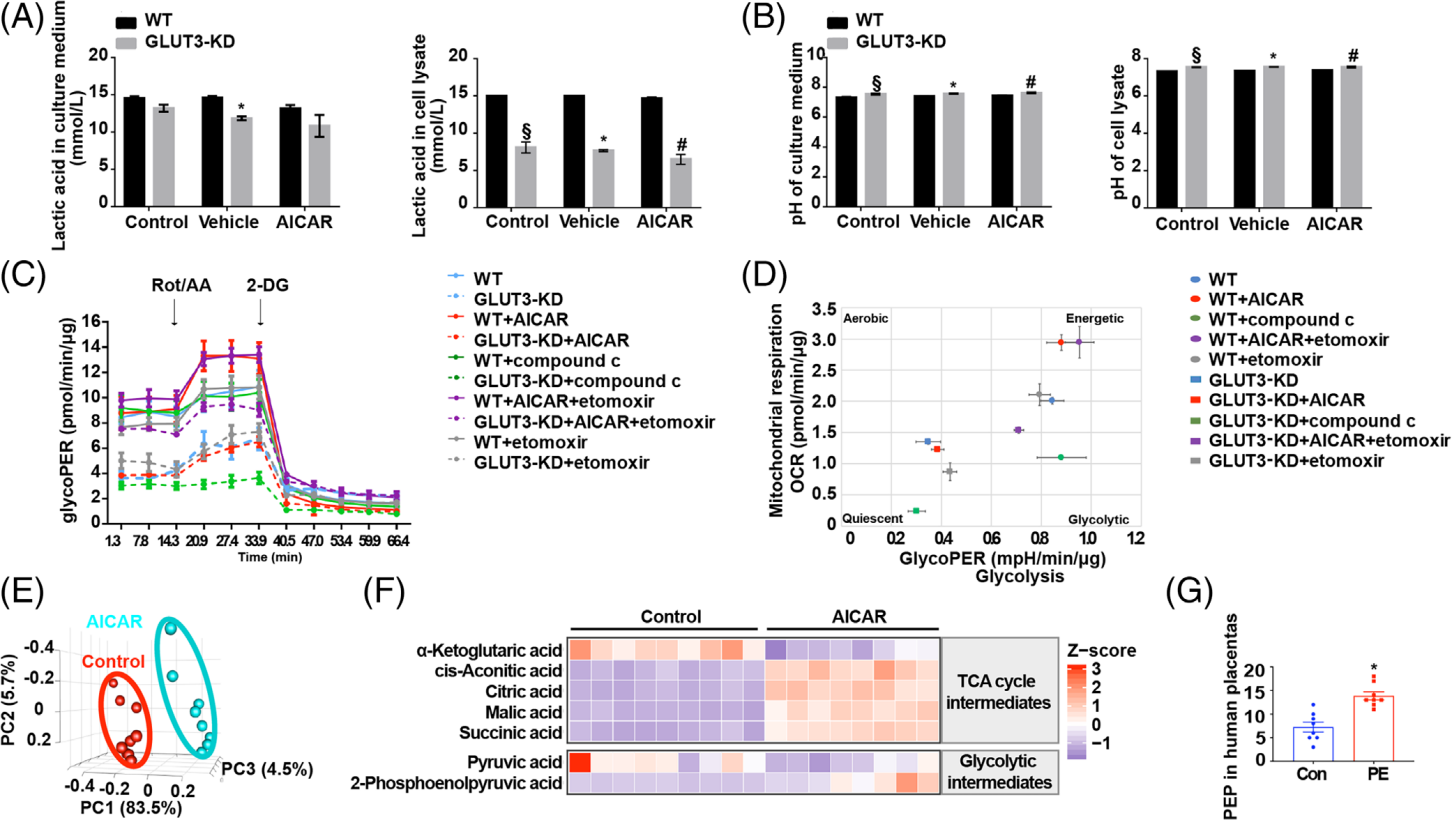
AMPK activation reprograms glucose metabolism, resulting in the accumulation of the glycolysis metabolite PEP. (A) Lactic acid and (B) pH levels in the cell culture medium and lysate of HTR8/SVneo cells after incubation with 200 μM AICAR for 24 h. *n* = 3; two‐way ANOVA and Tukey's multiple comparison test; *p < 0.05 versus WT vehicle, §*p* < 0.05 versus WT control, #*p* < 0.05 versus WT AICAR. (C) GlycoPERs of WT and shSLC2A3‐transfected cells treated with 200 μM AICAR, 10 μM compound C, 50 μM etomoxir or 200 μM AICAR and 50 μM etomoxir. (D) Baseline energy phenotypes of WT and sh*SLC2A3*‐transfected cells treated with 200 μM AICAR, 10 μM compound C, 50 μM etomoxir or 200 μM AICAR and 50 μM etomoxir. (E) Principal component analysis (PCA) of metabolites identified from control and 200 μM AICAR‐treated HTR8/SVneo cells. Principal component (PC) 1, PC2 and PC3 explained 83.5%, 5.7% and 4.5% of the variance, respectively. (F) Heatmap of the altered TCA cycle intermediates and glycolytic intermediates in HTR8/SVneo cells after 24 h of treatment with 200 μM AICAR. Red blocks indicate higher enrichment, whereas purple blocks represent lower enrichment. Only metabolites with p values less than 0.05 are shown. (G) PEP levels in human term placentas as detected by GC–MS. *n* = 8; two‐tailed *t*‐test; *p < 0.05. All data are presented as the mean ± SEM

How glucose uptake by GLUT3 is catabolized by AMPK hyperactivation was next investigated. Key enzymes of glycolysis, including hexokinase (HK) and phosphofructokinase (PFK), were upregulated in PE placentas, implying enhanced glycolysis, while lactate dehydrogenase (LDH) did not differ between the two groups (Figure S6A). Our in vitro data suggested that AICAR improved glucose uptake, but it did not result in increased lactate production in either WT or GLUT3‐KD HTR8/SVneo cells (Figure 8A). Furthermore, the pH of the cell lysate and culture medium was unchanged by AMPK activation in both groups of cells (Figure 8B). However, a significant decrease in intracellular lactic acid was observed in GLUT3‐KD cells compared with WT control cells; the amount of lactic acid in the culture medium was also slightly decreased in the GLUT3‐KD group. In accordance with this reduction in lactate production, both extracellular and intracellular pH were significantly higher for GLUT3‐KD cells than for WT cells (Figure 8B). These results suggest that although a notable portion of glucose uptake by GLUT3 is metabolized through the lactate pathway under basal conditions, glucose uptake via GLUT3 in trophoblasts is not metabolized to lactate due to AMPK hyperactivation.

### 3.8 GLUT3 is required for metabolic potential in response to AMPK activation in trophoblasts

The impact of the AMPK‐GLUT3 axis on trophoblast metabolism was further determined using a Seahorse XF Analyser. Generally, GLUT3‐KD cells exhibited a significantly lower OCR than WT cells (Figure S6B). Consistent with the lactic acid measurement results, GLUT3‐KD cells demonstrated a significantly lower glycolic proton efflux rate (glycoPER) than WT cells under basal conditions. AMPK activation with AICAR did not improve glycoPER in either group (Figures 8C,D and S6C,D). Nevertheless, AICAR significantly improved the OCR in WT cells, and this effect was largely attenuated in GLUT3‐KD cells (Figures 8C,D and S6B). However, inhibition of AMPK with compound C reduced the OCR in WT cells, while compound C treatment led to a complete shutdown of mitochondrial respiration in GLUT3‐KD cells. Moreover, inhibition of fatty acid β‐oxidation with etomoxir maintained the OCR and glycoPER in WT cells (Figures 8C, 6D, S6B,C). These findings reveal that trophoblasts are fueled predominantly by glucose through aerobic glycolysis rather than oxidative phosphorylation of fatty acids. Most importantly, although AMPK activation in trophoblasts elevates glucose utilization, it does not lead to lactate production. Additionally, GLUT3 is essential for the metabolic potential regulated by AMPK.

### 3.9 PEP, the production of which is induced by AMPK activation, preserves viability but suppresses invasion and migration

**FIGURE 9:**
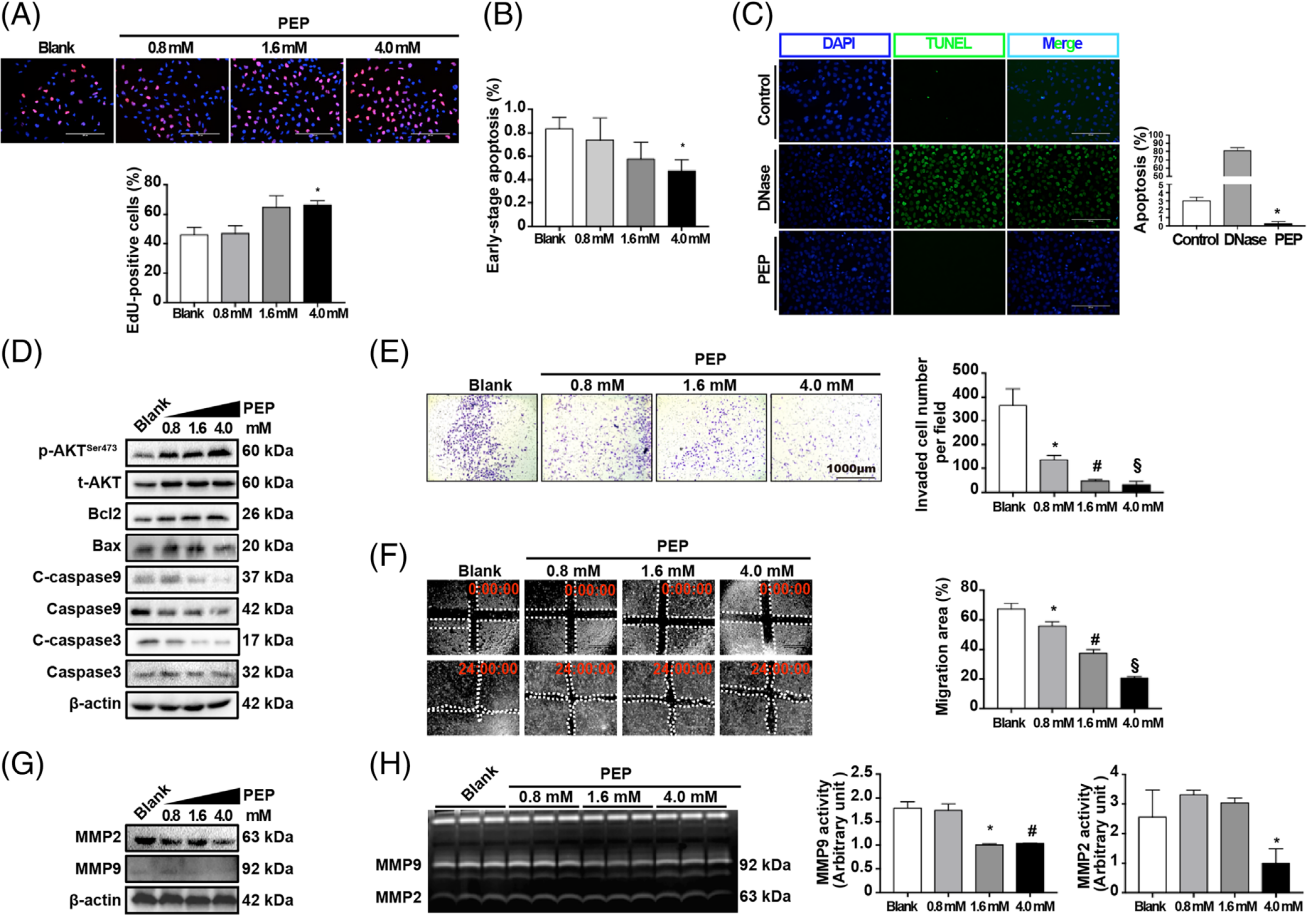
PEP functions as a double‐edged sword that benefits trophoblast survival and hinders its invasion. (A) Representative images and quantification of EdU staining of HTR8/SVneo cells after treatment with blank or with 0.8, 1.6 or 4.0 mM PEP. *n* = 3; one‐way ANOVA and Tukey's multiple comparison test; *p < 0.05 versus blank. (B) Apoptotic trophoblasts of the aforementioned four groups of cells were stained with Annexin V and measured by flow cytometry. *n* = 3; one‐way ANOVA and Tukey's multiple comparison test; *p < 0.05 versus blank. (C) Representative images and quantification of TUNEL staining in HTR8/SVneo cells subjected to different treatments. Blank controls were included. DNase treatment was used as a positive control. *n* = 3; one‐way ANOVA and Tukey's multiple comparison test; *p < 0.05 versus control. (D) Western blots of p‐AKT, t‐AKT, Bcl2, Bax, C‐Caspase9, Caspase 9, C‐Caspase3 and Caspase 3 in cells treated with blank or with 0.8, 1.6 or 4 mM PEP. β‐Actin was blotted as a loading control. (E) HTR8/SVneo cells were subjected to Matrigel Transwell assays in the presence of 0.8, 1.6 or 4.0 mM PEP. The invaded cells were stained and counted after 24 h of treatment. Blank controls were included. *n* = 3; one‐way ANOVA and Tukey's multiple comparison test; *p < 0.05 versus blank, #*p* < 0.05 versus blank, §*p* < 0.05 versus blank. (F) Wound‐healing assays for cells in the presence of 0.8, 1.6 or 4.0 mM PEP. Blank controls were included. The images were obtained after 0 and 24 h of treatment. The quantified migration areas are shown in the bar graph. *n* = 3; one‐way ANOVA and Tukey's multiple comparison test; *p < 0.05 versus blank; #*p* < 0.05 versus blank; §*p* < 0.05 versus blank. (G) Western blots of MMP2 and MMP9 in cells treated with blank or with 0.8, 1.6 or 4.0 mM PEP. β‐Actin was blotted as a loading control. (H) Gelatin zymography of MMP‐2 and MMP‐9 in the culture medium of HTR8/SVneo cells treated with blank or with 0.8, 1.6 or 4.0 mM PEP for 24 h. *n* = 3; one‐way ANOVA and Tukey's multiple comparison test; *p < 0.05 versus blank, #*p* < 0.05 versus blank. All data are presented as the mean ± SEM

To further trace glucose metabolism, the effects of AMPK on the bioenergetic profiles of trophoblasts were assessed by GC–MS, and AICAR‐treated HTR8/SVneo cells demonstrated a metabolome distinct from that of control cells (Figure 8E). Consistent with the observation in the Seahorse analysis, the levels of four intermediates, namely, cis‐aconitic acid, citric acid, malic acid and succinic acid, were found to be significantly elevated in the AICAR treatment group, which indicated that the tricarboxylic acid (TCA) cycle was enhanced (Figure 8F). Moreover, pyruvic acid levels significantly decreased with AICAR treatment, while PEP accumulated in AICAR‐treated trophoblasts. These results demonstrate that pyruvic acid generated from aerobic glycolysis due to AMPK hyperactivation is not only metabolized through oxidative phosphorylation but also redirected into gluconeogenesis and possibly ultimately glycogenesis and/or the pentose phosphate pathway (PPP). We also assessed the PEP levels in placentas from normal women and women with PE by using GC–MS. In accordance with our hypothesis, PEP levels and PEP carboxykinase (PEPCK) protein levels were increased in PE placentas (Figure 8G). Beyond its role in trophoblasts, PEPCK, which is a key enzyme responsible for PEP production from acetyl‐CoA, has been found to promote cancer cell survival.^42^ To determine whether PEP is the AMPK downstream effector that regulates trophoblast viability and invasion, various doses of PEP were applied to HTR8/SVneo cells. The results showed that PEP significantly enhanced proliferation (Figure 9A) but prohibited apoptosis of trophoblasts in a dose‐dependent manner (Figure 9B). Such antiapoptotic effects of PEP on trophoblasts were further confirmed by TUNEL staining (Figure 9C). PEP significantly elevated p‐AKT and Bcl2 levels but suppressed Bax2 levels and cleavage of caspases (Figure 9D). Moreover, PEP dose‐dependently compromised invasion and migration (Figure 9E,F). In support of this finding, the expression of MMP2 and MMP9 was downregulated by PEP treatment (Figure 9G). Additionally, MMP activity was repressed by PEP (Figure 9H). Altogether, our findings provide evidence that PEP, a glucose metabolite, may regulate trophoblast biological functions by preserving viability while suppressing invasion and migration.

## 4 DISCUSSION

PE has long been assumed to be an ischemia‐induced metabolic disorder of the placenta. This work reveals for the first time that PE placentas are not energy‐restricted. In addition to alterations in fatty acid metabolism identified in our early studies,^34^ a significant elevation in AMPK phosphorylation at Thr172 may have other pathogenic mechanisms. The upstream regulator of AMPK in the placenta is still inconclusive. AMPK can be activated by phosphorylation at Thr172 in the α subunit by upstream kinases,^43^, ^44^ and our current data suggest that LKB1 may be responsible for AMPK activation in PE placentas. LKB1 is a tumour suppressor protein, and inactivation of LKB1 under low‐energy conditions is a frequent event in various human cancers.^45^ Given that trophoblasts share numerous similarities with cancer cells,^21^ hyperactivation of LKB1, which may be caused by perturbations in sex hormones during pregnancy,^46^ is likely an unfavourable status for invading trophoblasts.

Incidentally, experiments ruling out the impacts of cell death and proliferation on invasion elucidated that the levels of the “survival signal” AKT^47^, ^48^ and the antiapoptotic protein Bcl2^49^ were enhanced by AICAR but suppressed by compound C, while those of the proapoptotic protein Bax changed inversely. Moreover, first‐trimester human villi, which contain aggressive EVTs, exhibited lower levels of p‐AMPK and prosurvival molecules than human term placentas, whereas human term placentas, in which trophoblastic invasion is largely lost, exhibited higher AMPK activity and elevations in anti‐apoptotic signalling. Based on our observations, we propose a balancing mechanism between “go” and “grow” in trophoblasts that is regulated by AMPK. This mechanism distinguishes trophoblasts from malignant tumour cells and, therefore, provides an innate regulatory machinery for the fate of trophoblasts. Although it prevents excessive invasion of trophoblasts into the uterus by elevating apoptosis, it compromises invasiveness to ensure trophoblast viability to maintain the integrity and basic functions of the placenta under conditions of stress.

In this study, we found that RUPP‐induced PE manifestations were potentiated by placental AMPK activation before RUPP surgery but partially relieved if AMPK was activated after placentation. This result is consistent with the previous report of Banek et al., which demonstrated that AICAR administration after RUPP ameliorates hypertension^50^ in rats. The protective effects of AMPK activation against PE are probably largely derived from its regulatory functions on trophoblast viability, angiogenesis and the nitric oxide pathway,^51^, ^52^, ^53^ rather than on trophoblast invasion. Our data are the first to provide in vivo evidence that AMPK activation at different times results in opposite effects in PE; therefore, placental AMPK should be carefully targeted in a tailored manner based on the desired effect.

We reported that activation of AMPK increases glucose uptake in trophoblasts, which is in accordance with previous findings in other types of cells,^54^, ^55^, ^56^ by specifically enhancing GLUT3 trafficking from the cytosol to the plasma membrane. GLUT3 recognizes and transports both α‐ and β‐d‐glucose anomers,^57^ which may contribute to a higher affinity and greater glucose transport capacity of GLUT3 than other GLUTs.^58^ Here, we obtained evidence that glucose transport via GLUT3 into HTR8/SVneo cells is comparable to that via GLUT1, which is consistent with previous findings in BeWo cells that GLUT3‐mediated glucose uptake accounts for half of the total glucose uptake.^59^ In addition, we found that GLUT3 deficiency significantly reduced glycoPER and OCR in HTR8/SVneo cells, which suggests that glucose is the main substrate of energy metabolism and that GLUT3 is the predominant GLUT in trophoblasts. Surprisingly, the regulation of trophoblast invasion and viability by AMPK was largely blunted by GLUT3 KD. Moreover, GLUT3 KD promoted invasion, exacerbated apoptosis and suppressed proliferation in trophoblasts. Given that previous findings have shown that increased enzyme activity of MMPs enhances the invasive activities in HTR8/SVneo cells,^60^ this study suggests that AMPK regulates trophoblast invasion by modulating MMPs. Additionally, the inhibitory effect of AMPK on MMPs was largely blunted by GLUT3 downregulation. Thus, our work elucidates a novel AMPK‐GLUT3 regulatory signalling pathway for trophoblast invasion.

It has been reported that trophoblasts exhibit substantial aerobic glycolysis^61^; however, neither PE placentas nor AICAR‐treated trophoblasts exhibit elevated lactic acid production. These results are consistent with cell viability data showing that AMPK activation protects against trophoblast death; otherwise, overproduction of lactic acid leads to acidosis and eventually causes cell death. However, although an enhanced TCA flux was induced in trophoblasts by AMPK activation, we also observed downregulation of IDH in PE‐complicated human placentas (Figure S6A). This finding indicates that the TCA improvement due to AMPK hyperactivation is well confined in vivo, possibly by feedback inhibition from the accumulated intermediates as well as increased ATP levels. Moreover, α‐ketoglutaric acid levels were significantly reduced in the presence of AICAR. Considering that α‐ketoglutaric acid is an important precursor of glutamate, a common amino donor, it may be largely consumed for biosynthesis by proliferating cells.

Intriguingly, similar to the accumulation of PEP in human PE placentas, significant elevations in PEP levels were observed in HTR8/SVneo cells after AICAR treatment. In addition to reductions in pyruvate levels, significant increases in malic acid levels were observed, while oxaloacetate remained unchanged, indicating that excessive glycolysis/TCA intermediates due to AMPK hyperactivation may be redirected into gluconeogenesis. Evidence from breast cancer cells has shown that knockdown of PEPCK, which converts oxaloacetate to PEP, reduces cell growth and increases cell death under stress conditions,^42^ suggesting that PEP may play a prosurvival role in trophoblasts. The beneficial effect of AMPK in sustaining the viability of trophoblasts may also be due to replenishment of molecules for cell proliferation through the polyol pathway and PPP^61^ as well as to AMPK‐mediated activation of Akt.^62^ Moreover, our metabolomics data showed significantly elevated NADP(H) levels in PE placentas, indicating stimulation of the PPP, which also generates ribose 5‐phosphate for nucleotide synthesis. Moreover, glycogen storage was observed (Figure S6E), which is consistent with the lack of glycogen and glyT in the placentas of *AMPKα1* ^*f/f*^ *Ada*‐Cre^+^ mice. These facts imply that AMPK hyperactivation increased PEP production from pyruvate/oxaloacetate and that PEP may be further used for glycogenesis and/or metabolized through the PPP to fulfil the demand for proliferation.

## 5 CONCLUSIONS

PE is associated with trophoblast AMPK hyperactivation, presumably due to LKB1 phosphorylation, and glucose uptake is consequently increased by trafficking GLUT3 from the cytosol to the plasma membrane. Such translocation enhances glycolytic flux and redirects glucose metabolic intermediates into gluconeogenesis, resulting in PEP accumulation, which not only benefits cell survival but also suppresses invasion by repressing MMPs. Our findings highlight the importance of AMPK‐mediated energy metabolism rewiring through GLUT3 in the regulation of trophoblast invasion and viability, thus providing in‐depth insight into trophoblast fate determination as well as the aetiology of PE from the perspective of glucose metabolism.

## AUTHOR CONTRIBUTIONS

Chao Tong conceived and designed the study; Li Wen, Jiayu Huang and Huijia Fu collected clinical samples; Baozhen Zhang and Xiujun Fan synthesized the nanoparticles; Ping Xu, Yangxi Zheng, Jiujiang Liao, Mingyu Hu, Yike Yang, Yamin Liu, Fumei Zhang, Xiyao Liu, Huili Jin, Liling Xiong and Yue Wu performed the animal and cell experiments; Tingli Han performed metabolomics; Rufei Gao and Mark D. Kilby interpreted the results; Ping Xu and Chao Tong wrote the draft; Ping Xu prepared the figures; Yong Fu and Philip N. Baker edited the manuscript; Chao Tong and Hongbo Qi provided funding; and Chao Tong, Philip N. Baker and Hongbo Qi cosupervised this work.

## FUNDING INFORMATION

This work was supported by the National Key R&D Program of China (2022YFC2702400), National Natural Science Foundation of China (81671488, 81871189, 82071675, U21A20346 and 82171662) and the Strategic Collaborative Research Program of the Ferring Institute of Reproductive Medicine.

## CONFLICT OF INTEREST

The authors declare that they have no competing interests.

## Supporting information


**Appendix S1** Supporting Information.

## Data Availability

Data and materials described in the manuscript will be available upon reasonable request to the corresponding authors; delivery charges and a material transfer agreement may apply.
