# Supplementary material for: AMPK regulates homeostasis of invasion and viability in trophoblasts by redirecting glucose metabolism: Implications for pre‐eclampsia
Source: Cell Prolif. 2022 Dec 8;56(2):e13358. doi: 10.1111/cpr.13358 (PMC9890534; doi:10.1111/cpr.13358)
Supplement: Supplementary file 1 — Appendix S1 Supporting Information. [file CPR-56-e13358-s001.docx]

**Supplementary Figures**


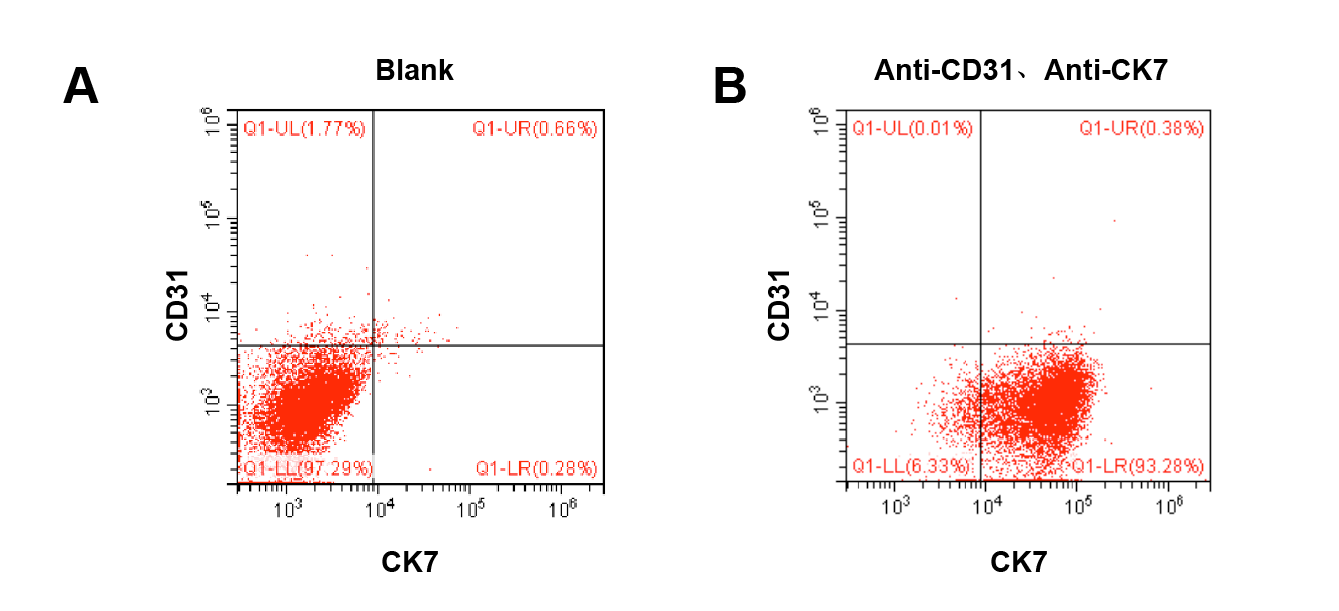


**Fig S1. Isolation of primary human trophoblasts (PHTs).**

Flow cytometry identification of isolated PHTs (A) without antibody labeling and (B) with anti-CD31 and anti-CK7 antibody labeling.


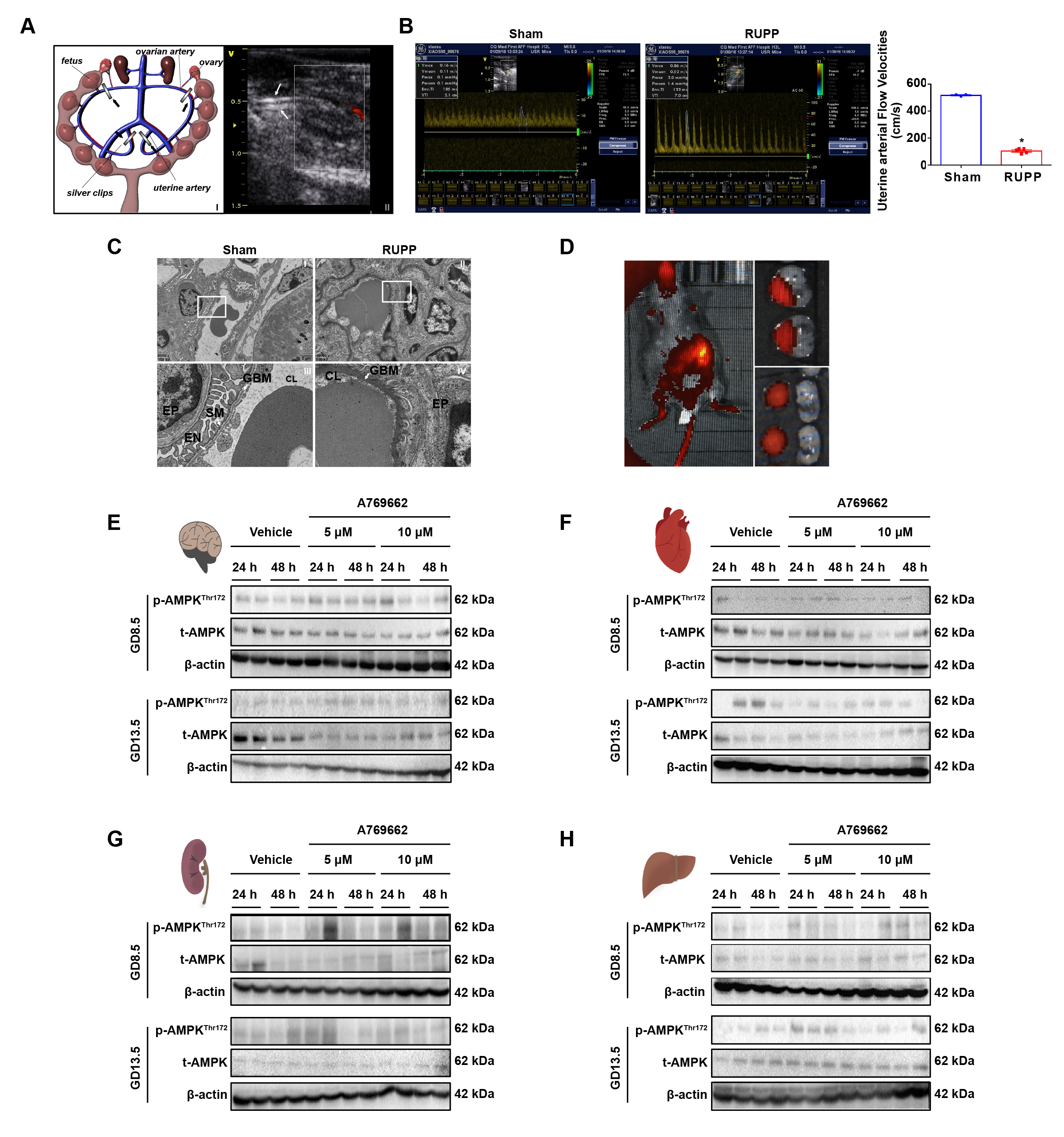


**Fig S2. RUPP-induced mouse PE model.**

(A) A PE mouse model was generated via RUPP. I. Schematic diagram of RUPP; II. silver clips (arrows) placed during laparotomy; III. Silver clip (arrow) observed via Doppler in living mice. (B) The hemodynamics of sham and RUPP dams were measured via Doppler. The ovarian and uterine arterial flow velocities were calculated. Sham n=3, RUPP n=5; two-tailed t test; *p<0.05. (C) Electron microscopy (EM) images of kidney tissue from sham (I, III) and RUPP mice (II, IV). Abbreviations in the graph: EN, fenestra of endothelial cells; GBM, glomerular basement membrane; SM, slit membrane; CL, capillary lumen; EP, podocyte. (D) At GD 13.5, pregnant mice were injected with plCSA-NPs via the tail vein and imaged with an IVIS spectrum optical imaging system. A769662 (5 μM or 10 μM) loaded in plCSA-NPs was injected into pregnant mice at either GD 8.5 or GD 13.5, and the levels of AMPK activation in the (E) brain, (F) heart, (G) kidneys and (H) liver were determined via Western blotting.

**
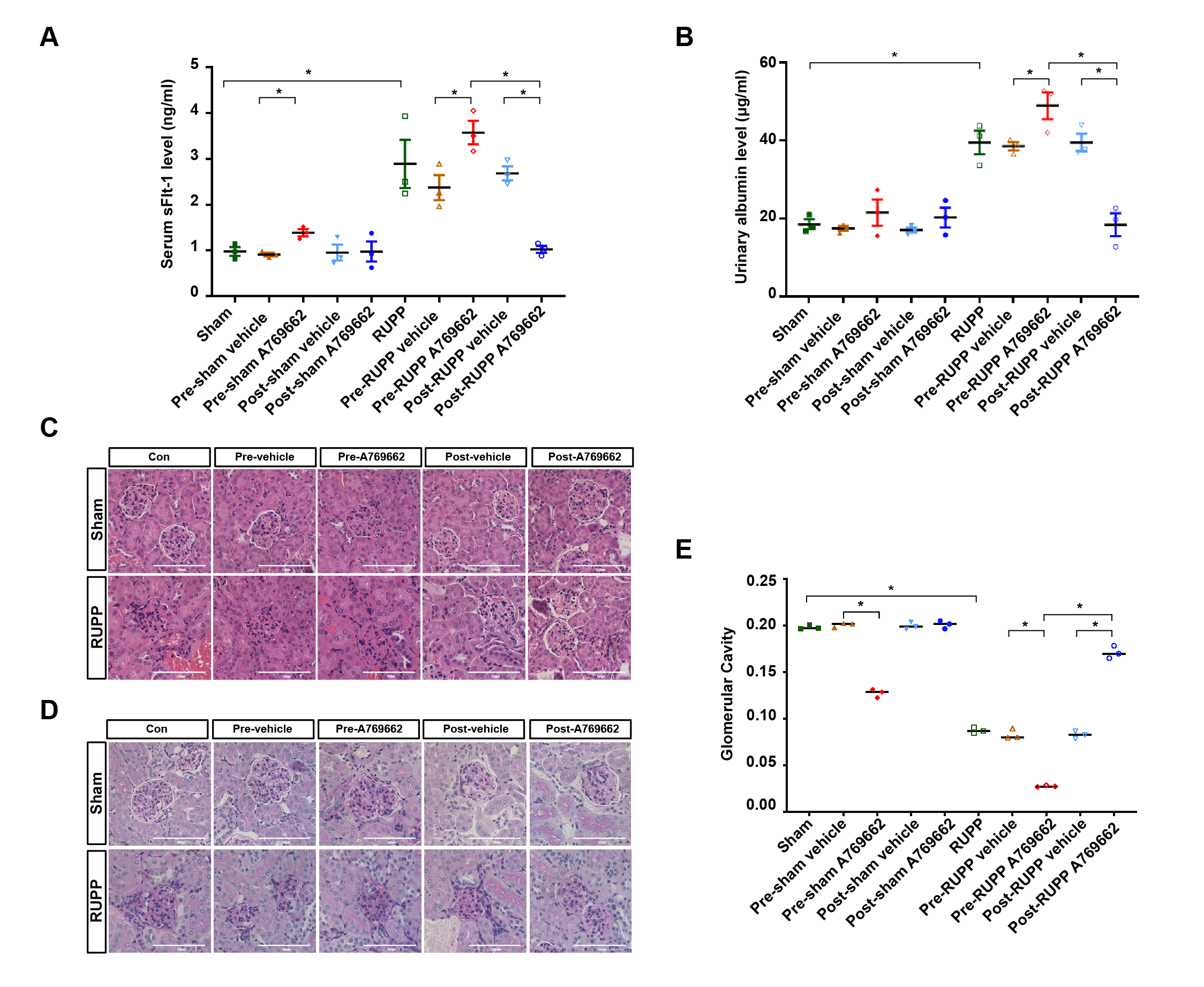
 Fig S3 Activation of placental AMPK during placentation exacerbated RUPP-related serum sFlt-1 induction and kidney damage.**

(A) The levels of serum sFlt-1 were detected in samples from RUPP or sham mice treated with 5 μM A769662 or vehicle loaded in plCSA-NPs at either GD 8.5 or immediately after operation. n=3; one-way ANOVA and Tukey’s multiple comparison test; *p<0.05. (B) Urinary albumin was measured. n=3; one-way ANOVA and Tukey’s multiple comparison test; *p<0.05, #p<0.05, §p<0.05, ∆p<0.05. (C-D) Representative H&E staining (C) and PAS staining (D) of paraffin sections of kidney tissue. (E) Glomerular cavities were measured as the ratio of Bowman’s capsule to renal corpuscle area by using ImageJ 1.50i software. n=3; one-way ANOVA and Tukey’s multiple comparison test; *p<0.05. All data are presented as the mean ± SEM.

**
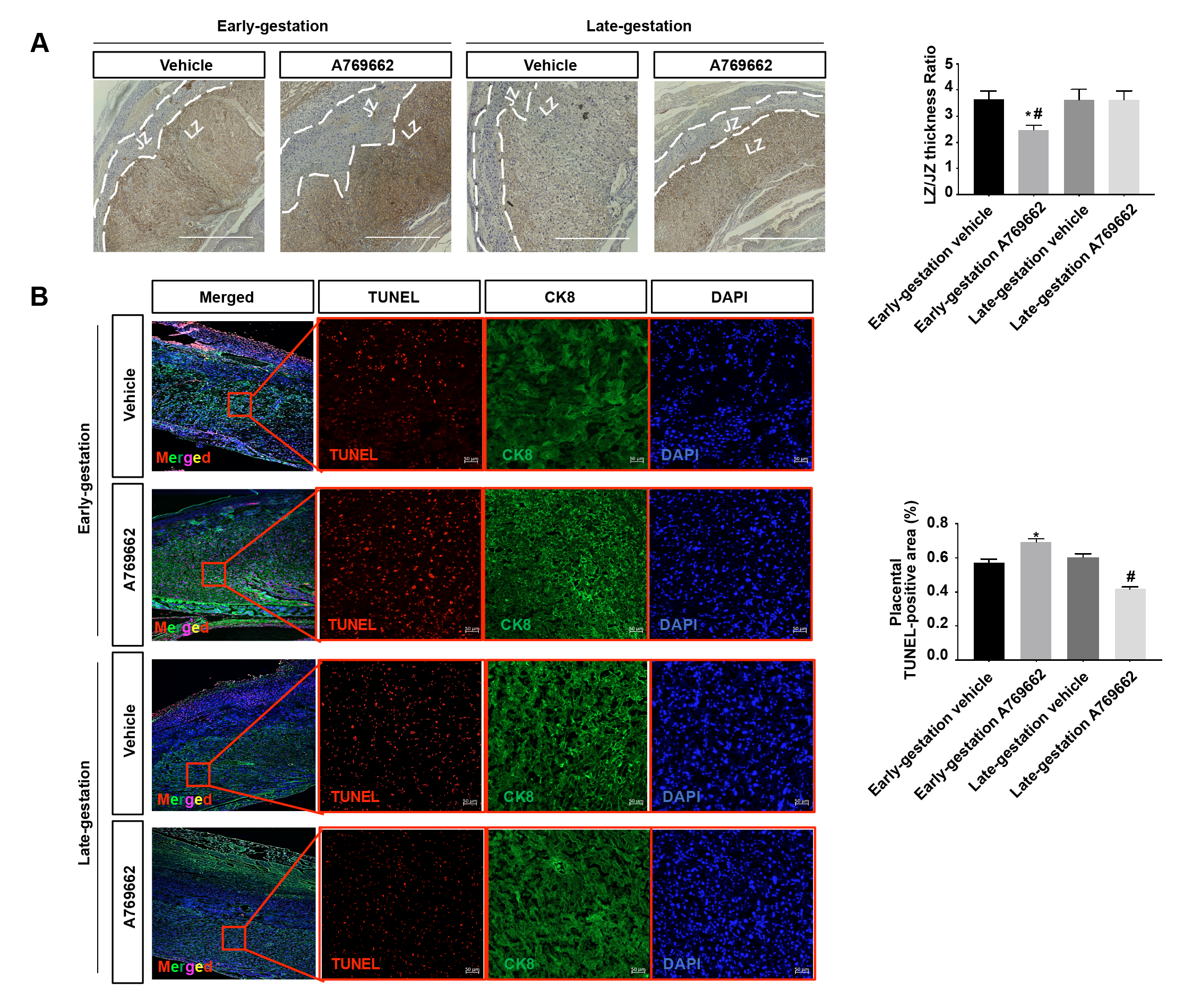
 Fig S4 Activation of placental AMPK during placentation impaired placental development and subsequently induced TUNEL signals.**

(A) The LZ/JZ thickness ratio was detected at GD 18.5. n=3; one-way ANOVA and Tukey’s multiple comparison test; *p<0.05 vs. vehicle at early gestation, #p<0.05 vs. A769662 at late gestation. (B) Representative images and quantification of TUNEL staining in different treatment groups. n=3; one-way ANOVA and Tukey’s multiple comparison test; *p<0.05 vs. early gestation-vehicle, #p≤0.05 vs. late gestation-vehicle. All data are presented as the mean ± SEM.

**
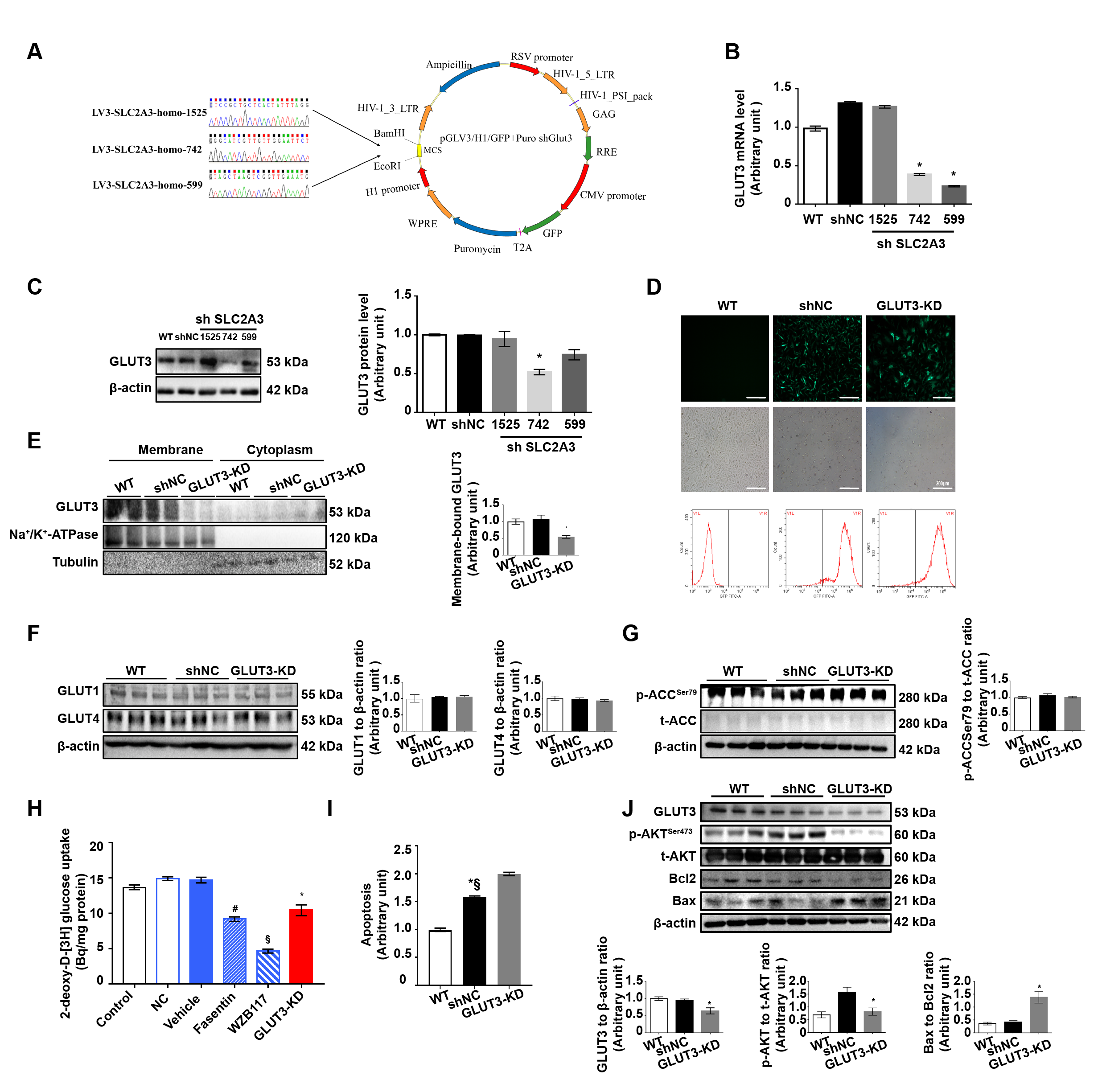
**

**Fig S5 GLUT3 KD downregulated GLUT3 in HTR8/SVneo cells.**

(A) Map of 3 shRNAs targeting the GLUT3-encoding gene SLC2A3. (B) GLUT3 mRNA levels were determined by RT‒PCR. n=3; one-way ANOVA and Tukey’s multiple comparison test; *p<0.05 vs. shNC. (C) GLUT3 protein levels were determined by Western blotting. n=3; one-way ANOVA and Tukey’s multiple comparison test; *p<0.05 vs. shNC. (D) After transfection, GFP-tagged shRNA was visualized with a fluorescence microscope, and the results were further verified by flow cytometry. (E) GLUT3 expression in membrane and cytoplasmic fractions from untransfected, shNC-transfected and shSLC2A3-transfected HTR8/SVneo cells was evaluated by Western blotting. Tubulin and Na^+^/K^+^ ATPase were blotted as loading controls. n=3; one-way ANOVA and Tukey’s multiple comparison test; *p<0.05 vs. shNC. (F) Western blots of GLUT1 and GLUT4 in the aforementioned 3 groups of HTR8/SVneo cells. β-Actin was used as a loading control. n=3; one-way ANOVA and Tukey’s multiple comparison test. (G) Western blots of P-ACC and t-ACC in the aforementioned 3 groups of HTR8/SVneo cells. β-Actin was used as a loading control. n=3; one-way ANOVA and Tukey’s multiple comparison test. (H) 2-Deoxy-D-[^3^H] glucose uptake into HTR8/SVneo cells after 24 hours of treatment with 100 μM fasentin or 10 μM WZB117 or transfection with shNC or shSLC2A3. Controls were included. n=3; one-way ANOVA and Tukey’s multiple comparison test; *p<0.05 vs. shNC, §p<0.05 vs. vehicle, #p<0.05 vs. vehicle. (I) Apoptotic trophoblasts were stained with Annexin V and measured by flow cytometry. n=3; one-way ANOVA and Tukey’s multiple comparison test; *p<0.05 vs. WT, §p<0.05 vs. shNC. (J) Western blots of GLUT-3, p-AKT, t-AKT, Bcl2, and Bax; β-actin was blotted as the loading control. n=3; one-way ANOVA and Tukey’s multiple comparison test; *p<0.05 vs. shNC. All data are presented as the mean ± SEM.


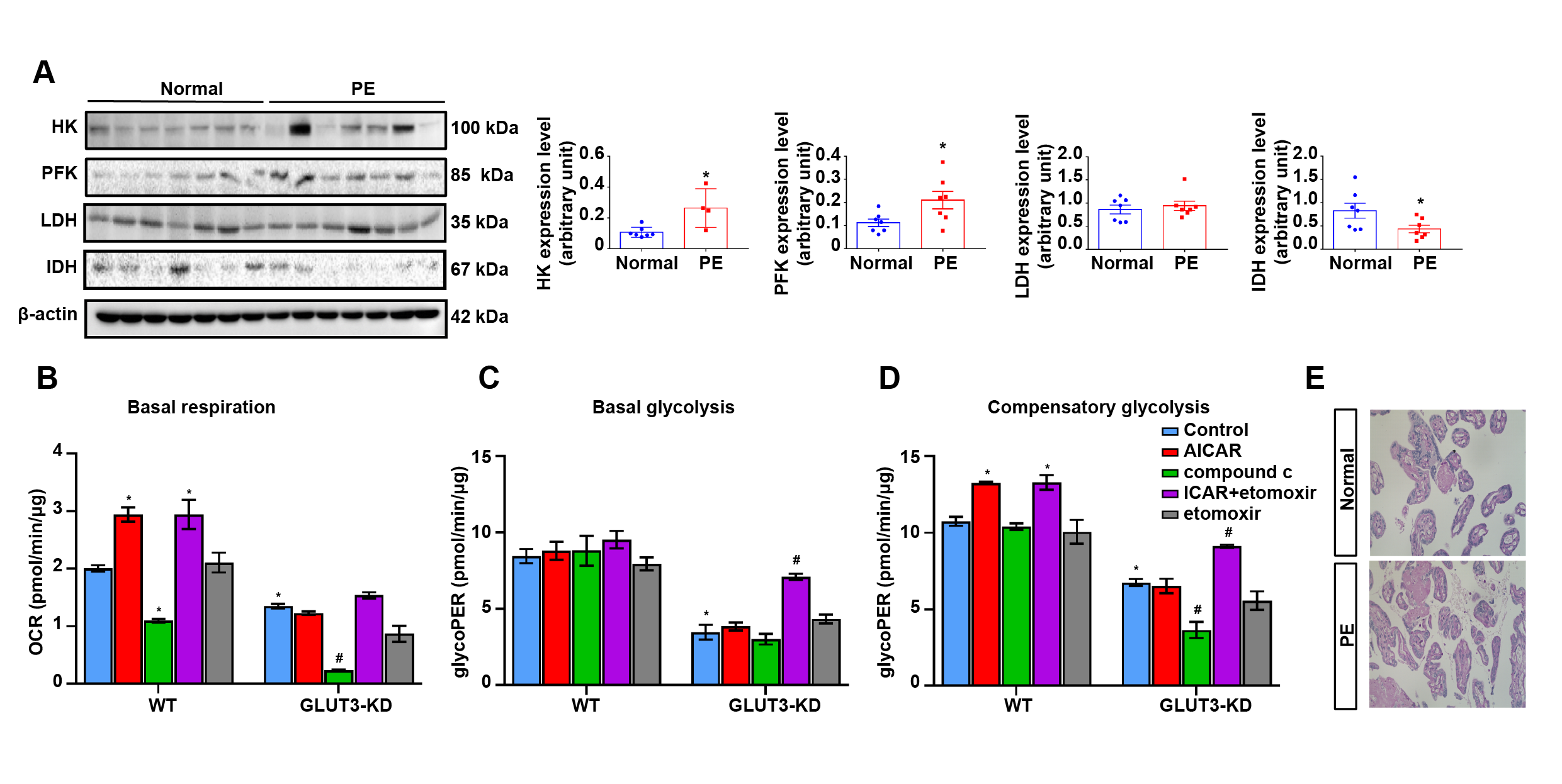


**Fig S6 AMPK activation reprogrammed glucose metabolism in trophoblasts.**

(A) Western blotting of HK, PFK, LDH, and IDH in normal and PE-complicated placentas. β-Actin was used as a loading control. n=7; two-tailed t test; *p<0.05. (B) Basal respiration rates of WT and GLUT3-KD cells in the presence of 200 μM AICAR, 10 μM compound C, 50 μM etomoxir or 200 μM AICAR and 50 μM etomoxir as calculated from the OCRs. n=3; two-way ANOVA and Tukey’s multiple comparison test; *p<0.05 vs. WT control, #p<0.05 vs. GLUT3-KD control. (C) Basal glycolysis of WT and GLUT3-KD cells in the presence of 200 μM AICAR, 10 μM compound C, 50 μM etomoxir or 200 μM AICAR and 50 μM etomoxir as calculated from the glycoPERs. n=3-4; two-way ANOVA and Tukey’s multiple comparison test; *p<0.05 vs. WT control, #p<0.05 vs. GLUT3-KD control. (D) Compensatory glycolysis of the aforementioned groups of cells as calculated from the glycoPERs. n=3-4; two-way ANOVA and Tukey’s multiple comparison test; *p<0.05 vs. WT control, #p<0.05 vs. GLUT3-KD control. (E) PAS staining of normal and PE-complicated placentas. The arrows show the deposited glycogen. Scale bar, 200 μm. All data are presented as the mean ± SEM.

**Supplementary tables**

**Table S1. Clinical characteristics of the subjects.**

| Parameters | Control group  (n=15) | PE group  (n=15) | p value |
| --- | --- | --- | --- |
| Age (year) | 31.33 ± 0.95 | 30.33 ± 1.30 | 0.54 |
| Body Mass Index (pre-gestation) | 20.65 ± 0.67 | 22.59 ± 0.77 | 0.07 |
| Body Mass Index (prenatal) | 26.71 ± 0.73 | 28.31 ± 0.88 | 0.17 |
| Systolic Blood Pressure (mmHg) | 109.3 ± 2.18 | 164.4 ± 2.83 | < 0.05 |
| Diastolic Blood Pressure (mmHg) | 65.27 ± 1.33 | 100.9 ± 1.80 | < 0.05 |
| Gestational Age (week) | 39.62 ± 0.23 | 36.14 ± 0.58 | < 0.05 |
| Neonatal Birth Weight (gram) | 3343 ± 72.23 | 2925 ± 253.70 | 0.06 |
| Placental Weight (gram) | 540.1 ± 11.84 | 488.7 ± 25.03 | 0.07 |
| Fetal sex (male/female) | 8/7 | 8/7 | N/A |

**Table S2. Sequence of the shRNA targeting SLC2A3.**

| sh SLC2A3 | Sequence (5'to3') |
| --- | --- |
| LV3-SLC2A3-homo-1525 | CTCCGCTGCTCACTATTTAGG |
| LV3-SLC2A3-homo-742 | GGGCATCGTTGTTGGAATTCT |
| LV3-SLC2A3-homo-599 | GTAGCTAAGTCGGTTGAAATG |

**Table S3. Sequences of the primers for RT-qPCR.**

| Primer | Sequence |
| --- | --- |
| GLUT3-F | 5’-TCCCCTCCGCTGCTCACTATTT-3’ |
| GLUT3-R | 5’-ATCTCCATGACGCCGTCCTTTC-3’ |
| β-actin-F | 5’-TGGCACCCAGCACAATGAA-3’ |
| β-actin-R | 5’-CTA AGTCATAGTCCGCCTAGAAGCA-3’ |

**Table S4. Sequences of the genotyping primers.**

| Primer | Sequence |
| --- | --- |
| 5’LoxP-F | 5’-CATCTTCTGAGCGTTCTCTC-3’ |
| 5’LoxP-R | 5’-CAGTTGGGAGCCTGCTTGGC -3’ |
| 3’LoxP-F | 5’- GGAAGTAGACTTACAGGGCT-3’ |
| 3’LoxP-R | 5’-GAGAGACAGTGCGTAACATG-3’ |
| ADACre-F | 5’- TTGCTGTTGGCCATGTGAGGAGAC-3’ |
| ADACre-R | 5’- CAGCCCGGACCGACGATGAAGC-3’ |
